# Selenophene *π*‐Bridge Enables Balanced Ambipolar Transport in High‐Performance Organic Electrochemical Transistors

**DOI:** 10.1002/advs.76016

**Published:** 2026-06-09

**Authors:** Guichuan Zhu, Yueping Lai, Jiaxing Pu, Jianhua Chen, Liang‐Wen Feng

**Affiliations:** ^1^ Key Laboratory of Green Chemistry & Technology Ministry of Education College of Chemistry Sichuan University Chengdu China; ^2^ Department of Chemical Science and Technology Yunnan University Kunming China

**Keywords:** balanced single‐component ambipolar polymer, inverter, organic electrochemical transistors, selenophene *π*‐bridge

## Abstract

Developing single‐component ambipolar organic mixed ionic‐electronic conductors (OMIECs) is crucial for realizing low‐power complementary circuits and biosensors based on organic electrochemical transistors (OECTs). However, there is a shortage of high‐performance single‐component ambipolar OMIECs, along with the issue of performance mismatch between p‐type and n‐type. Here, we copolymerized diketopyrrolopyrrole with selenophene serving as the *π*‐bridge to synthesize a new ambipolar polymer, P(gTDPPSe). The selenophene *π*‐bridge endows the polymer with favorable doping energy levels and a narrow bandgap, enabling P(gTDPPSe) to possess balanced ambipolar transport. Vertical OECTs of P(gTDPPSe) were fabricated, which demonstrate balanced and remarkable performance in both p‐type and n‐type modes, achieving peak transconductance of 192.9 and 117.6 mS, respectively. Notably, the peak *I*_ON_ of 57.3 mA (*V*_DS_ = −0.5 V, *V*_G_ = −1.0 V) in p‐type mode is the highest among reported single‐component ambipolar materials. Building upon this, the P(gTDPPSe)‐based inverter achieved a maximum gain as high as 163 V/V. In this study, selenophene *π*‐bridge enables precise control over molecular energy levels, planarity, and packing, yielding a high‐performance balanced ambipolar copolymer. This presents a feasible approach to acquire balanced single‐component ambipolar materials.

## Graphical abstract

We synthesized a high‐performance balanced ambipolar OECT polymer, P(gTDPPSe), via selenophene *π*‐bridge engineering. The selenophene *π*‐bridge enhances backbone planarity and optimizes the energy levels for doping, enabling high and balanced p/n‐type transconductance‐a key factor for high‐gain complementary inverters. These features establish selenophene *π*‐bridge engineering as an effective strategy for developing balanced ambipolar OECT materials.

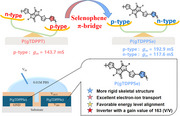

## 1 Introduction

Organic electrochemical transistors (OECTs) exhibit significant potential in advancing the diversification of bioelectronics, owing to their low operating voltage (typically < 1 V), high transconductance (>10 mS), excellent biocompatibility [1, 2, 3, 4, 5, 6, 7, 8, 9, 10]. OECT‐based inverters serve as the fundamental building blocks of complementary logic circuits, offering low power consumption, high reliability, and the capability for complex mixed‐signal processing and bioelectronic interfacing [11, 12, 13]. Compared with the approach of constructing p‐n organic complementary logic circuits using different n‐type and p‐type materials, single‐component ambipolar organic mixed ionic‐electronic conductors (OMIECs) significantly reduce process complexity and processing costs, while further enhancing organic electronics integrability [14]. Moreover, they could form a uniform morphology, avoiding issues like phase separation or heterogeneous doping, thus ensuring a higher yield and batch‐to‐batch reproducibility [15, 16, 17, 18, 19]. Additionally, balanced ambipolar transport ensures the equilibrium between hole and electron conduction, reduces charge injection barriers between the semiconductor and electrodes, and enables full‐swing voltage output, high gain, all of which are indispensable characteristics for high‐performance logic circuits and electrophysiological applications [20, 21]. Therefore, the development of balanced single‐component ambipolar OECTs holds significant importance.

An ideal single‐component ambipolar OMIECs typically needs to meet the following conditions: (i) the energy levels of the highest occupied molecular orbital (HOMO) and the lowest unoccupied molecular orbital (LUMO) of the materials should be well‐matched with the redox window to minimize the energy level differences with the gold electrode (with a work function of ≈ 5.1 eV) to enable efficient hole and electron transport [22, 23, 24]; (ii) incorporating polar functional groups to establish an ion‐permeable network [25, 26]; (iii) possessing appropriate crystallinity, crystal phase size, and roughness to enable efficient ionic‐electronic coupling [27]. Currently, the reported high‐performance single‐component ambipolar OMIECs are limited, with performance imbalance observed between the p‐type and n‐type [15, 28, 29] Therefore, we will further optimize the energy levels and carrier transport properties of the material by copolymerizing suitable electron‐donating (D) and electron‐accepting (A) units to synthesize new single‐component ambipolar OMIECs [30, 31].

Diketopyrrolopyrrole (DPP) is a versatile electron‐deficient building block, and numerous high‐performance p‐type organic semiconductors developed based on it have been reported [32, 33, 34, 35, 36]. Previous reports have demonstrated that *π*‐bridge engineering can effectively regulate molecular planarity, charge delocalization, and energy level alignment [37]. Compared with common thiophene *π*‐bridge, the selenophene *π*‐bridge has the following advantages: (1) the incorporation of selenophene *π*‐bridge could further enhance the packing of polymers, due to the metallic properties of selenium [38, 39]; (2) the lower aromaticity of the selenophene *π*‐bridge will facilitate the polymers to exhibit stronger quinone‐like resonance characteristics, expanding the region of electron delocalization [40, 41, 42]; (3) the selenophene *π*‐bridge induces a more planar backbone conformation, narrowing the bandgap and enhancing intermolecular packing, thereby promoting high charge carrier mobility [43, 44, 45, 46]; (4) increasing the selenophene content in polymer backbones can endow the metalloid with better hydrophilicity and higher volumetric capacitance, further enhancing the ionic‐electronic coupling capability of materials [47]. The introduction of selenophene into the polymer can further lower the LUMO level [45, 48], resulting in a smaller energy offset between it and the work function of the gold electrode [49]. This significantly improves the n‐doping efficiency of the material. Therefore, selenophene *π*‐bridge as a donor group was used to combine with DPP to construct a D‐A backbone for synthesizing single‐component ambipolar materials.

**FIGURE 1:**
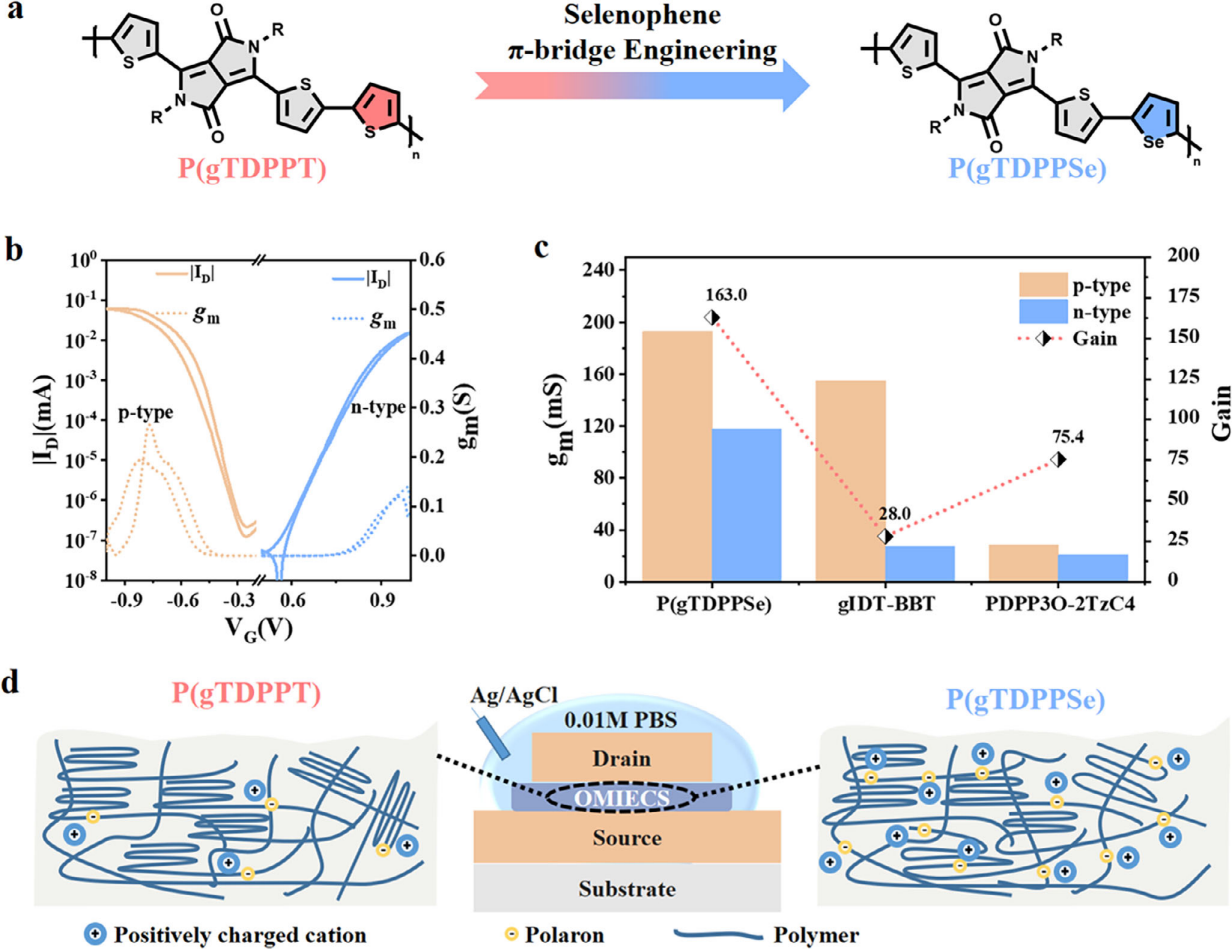
(a) Changes in the structure of P(gTDPPT) and P(gTDPPSe). (b) Ambipolar transfer curves of patterned P(gTDPPSe). (c) Summarized gain values and transconductance the inverters based on ambipolar OECTs. (d) Simulation diagram of the working conditions after n‐doping of two polymers.

Here, branched ethylene glycol DPP units were used to copolymerize with thiophene and selenophene, resulting in P(gTDPPT) and P(gTDPPSe), respectively (Figure 1a). Compared with P(gTDPPT), P(gTDPPSe) not only lowers the LUMO level but also effectively narrows the bandgap, allowing the energy levels of the semiconductor molecules to achieve smaller energy offset with the work function of gold (≈ 5.1 eV), which facilitates the injection of electrons from the gold electrode into the semiconductor. The theoretical calculations and microscopic structural characterization further indicate that the incorporation of selenophene *π*‐bridges facilitates the formation of intramolecular Se···H interactions, significantly enhancing charge mobility [50]. Meanwhile, testing with Electrochemical Impedance Spectroscopy showed that the presence of the selenophene bridge was beneficial for ion storage. The fabricated vertical OECTs (vOECTs) based on P(gTDPPSe) exhibited excellent ambipolar performance, achieving maximum drain currents *I*_ON_ of 57.3 and 14.0 mA, and maximum peak *g*_m_ of 192.9 and 117.6 mS in p‐type and n‐type modes, respectively (Figure 1b). The p‐type *I*_ON_ of 57.3 mA was more than 20% higher than the highest current reported for ambipolar OECTs [51]. Moreover, its p‐type performance also showed nearly a 34% increase in transconductance compared to P(gTDPPT). Meanwhile, we fabricated the inverters, and the maximum voltage gain of P(gTDPPSe) reached 163 V/V (Figure 1c), demonstrating outstanding performance among single‐component OECT‐based inverters [15, 17]. This indicates that if the p–n pair remains unbalanced, simply optimizing the individual performance of either p‐type or n‐type transistors fails to deliver high voltage gain in OECT inverters, as demonstrated in gIDT‐BTT [30]. In contrast, even if the individual transport characteristics of p‐type and n‐type channels are moderate, well‐balanced and synchronized operation can still yield a relatively high voltage gain, as demonstrated in PDPP3O‐2TzC4 [16]. It reveals that a high‐gain demands both balanced and high transconductance. In this study, we reported a high‐performance balanced ambipolar polymer synthesized via a selenophene *π*‐bridge strategy, which enabled high‐gain organic electrochemical inverters, thereby advancing the development of complementary logic circuits.

## 2 Results and Discussion

The detailed synthesis procedures of the monomers and copolymers can be found in supporting information [52]. We constructed two polymers, namely P(gTDPPT) and P(gTDPPSe), copolymers of DPP with thiophene and selenophene, respectively (Figure 1a). By synthesizing branched ethylene glycol side chains, excellent ion injection and extraction capabilities in aqueous environments and good solubility in chloroform were achieved for the DPP copolymers [53]. In addition, the obtained polymers were purified by consecutive Soxhlet extraction with methanol, acetone, and n‐hexane to remove low‐molecular‐weight components and impurities. The purified polymers were extracted with chloroform, precipitated with methanol, followed by filtration, and further dried under vacuum. Nuclear magnetic resonance (NMR) ^1^H and ^13^C were used to characterize the chemical structure (Supporting Information). The product polymers were obtained and characterized by gel‐permeation chromatography (room temperature, hexafluoroisopropanol as eluent), showing comparable number‐average molecular weights (*M*_n_s) and polydispersity indices (PDIs) of 13.1/1.76 and 15.4/1.94 kDa for P(gTDPPT) and P(gTDPPSe) (Figure S1). Thermogravimetric analysis (Figure S2) showed that they had high decomposition temperatures of >320°C, and further differential scanning calorimetry (Figure S3) in the temperature range of 50°C –250°C did not observe phase transitions or glass transitions.

**FIGURE 2:**
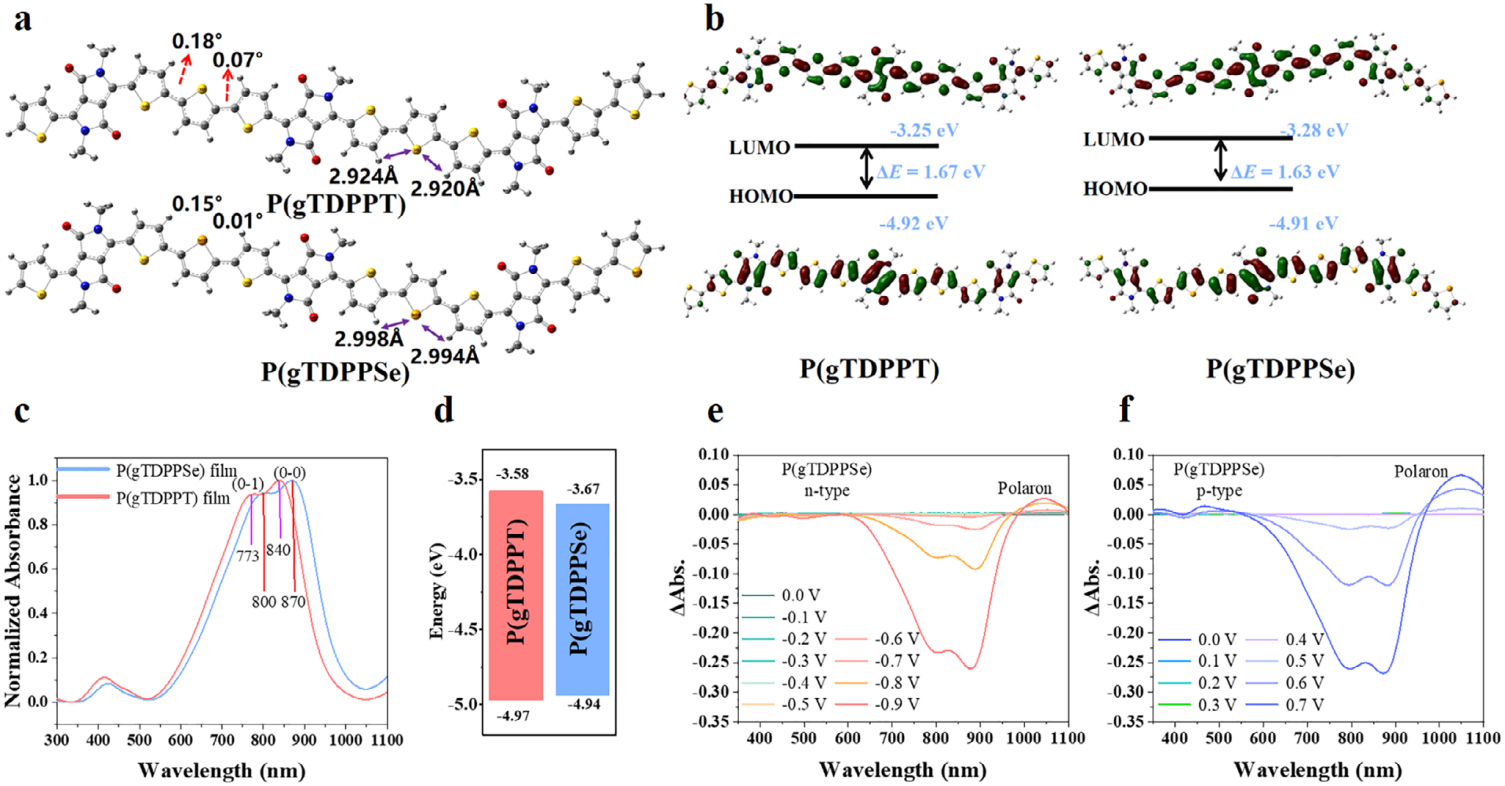
(a) The optimized molecular geometry. (b) Frontier molecular orbitals using DFT calculations at B3LYP‐D3BJ/def2‐SVP level. (c) Absorption spectra for patterned P(gTDPPSe) and patterned P(gTDPPT) as thin films. (d) HOMO and LUMO levels of P(gTDPPSe) and P(gTDPPT) measured by cyclic voltammetry in acetonitrile using 0.1 m tetrabutylammonium hexafluorophosphate (Bu_4_NPF_6_) as the supporting electrolyte. The evolution of the spectra of patterned P(gTDPPSe) film under reduced (e) and oxidized (f) states in 0.01 m Phosphate‐Buffered Saline (PBS) aqueous solution at various biasing conditions.

To comprehensively understand the geometric and electronic structures of the synthesized materials, density functional theory (DFT) calculations were performed based on the Gaussian method (B3LYP‐D3BJ/def2‐SVP). The branched ethylene glycol side chains were treated as methyl groups to simplify the calculations. Since OECTs materials typically operate in a highly doped state, the molecular properties in a highly doped state may significantly affect charge transport performance. Therefore, we calculated the properties of the two polymers’ doped states. The energy difference between the neutral and negatively charged state (*ΔE*  =  *E*_negative _− *E*_neutral_) of P(gTDPPT) was −60.675 kcal/mol, which is more negative than that of P(gTDPPSe) (−61.902 kcal/mol) in Figure S4. These results suggested that the negative polarons on P(TDPPSe) backbone constructed with a selenophene *π*‐bridge are more stable, and the stability is not related to the LUMO energy levels [53]. Furthermore, we also calculated the energy difference between the neutral and positively charged states of the two polymers. The results showed similar energy differences, indicating comparable p‐doping capabilities (Figure S4). The DFT calculations showed that P(gTDPPSe) has a lower negative‐charge‐state energy than P(gTDPPT), favoring n‐type doping of positively charged cation in vOECTs (Figure 1d), while keeping a comparable positive‐charge‐state energy for p‐type doping, indicating clear ambipolar doping potential. Later, we calculated the optimized geometries of two trimer structures and their frontier molecular orbitals, as shown in Figure 2a. In P(gTDPPT), the dihedral angles between the DPP units and adjacent acceptors are approximately 0.07° and 0.18°, while in P(gTDPPSe), the dihedral angles between the DPP units and adjacent acceptors are approximately 0.01° and 0.15°. The results showed that the trimer of the P(gTDPPSe) constructed with a selenophene *π*‐bridge exhibits a more planar conformation, promoting efficient charge transport. As shown in Figure 2a, the distance between the Se atom on the selenophene unit of P(gTDPPSe) and the adjacent hydrogen atoms were 2.998 and 2.994 Å, less than the sum of the van der Waals radii of the two atoms (1.900 and 1.200 Å, totaling 3.100 Å), indicating a strong covalent interaction along the backbone between Se···H [49]. Similarly, the distance between the S atom on the thiophene unit of polymer

P(gTDPPT) and adjacent hydrogen atoms is 2.924 and 2.920 Å, indicating that S···H also exhibits covalent interactions along the backbone [54]. However, compared to P(gTDPPT), the decrease in van der Waals distance between Se···H in P(gTDPPSe) is greater, suggesting that the intermolecular interactions of P(gTDPPSe) are stronger, and this also confirms that the selenophene *π*‐bridge in P(gTDPPSe) mentioned earlier contributes to its better planarity [55]. Overall, these results suggest that the selenophene *π*‐bridge facilitates excellent charge transport, enabling high‐performance ambipolar OECTs. In addition, the LUMO and the HOMO are shown in Figure 2b. Both trimers exhibit localized electronic structures, with the donor units mainly contributing to the HOMO, while the LUMO is primarily confined to the electron‐deficient DPP units. The *E*_LUMO_/*E*_HOMO_ values obtained from DFT simulations are −3.25/−4.92 eV for P(gTDPPT) and −3.28/−4.91 eV for P(gTDPPSe). From P(gTDPPT) to P(gTDPPSe), the LUMO level slightly decreases, which is particularly evident in the subsequent cyclic voltammetry tests.

Visible‐near infrared absorption measurements were performed to examine the optical characteristics of the patterned P(gTDPPT) and patterned P(gTDPPSe), as shown in Figure 2c and Figure S5 with the relevant data summarized in Table 1. P(gTDPPT) and P(gTDPPSe) exhibited similar absorption profiles in both solution and thin films, characterized by a dual‐band absorption. The absorption bands in the high‐energy regions, spanning from 335 to 510 nm (in solution) or 350 to 520 nm (in film), are attributed to *π*–*π* ^*^ transitions, while the low‐energy absorption bands, ranging from 510 to 950 nm (in solution) or 520 to 1050 nm (in film), correspond to intramolecular charge transfer (ICT). The maximum absorption peak of the P(gTDPPT) solution was at 804 nm, while the maximum absorption peak of the P(gTDPPSe) solution was at 830 nm, indicating that the absorption peak of the P(gTDPPSe) had undergone an overall redshift. This suggested that the P(gTDPPSe) exhibits a higher degree of aggregation compared to the polymer P(gTDPPT), which indicated that the selenophene *π*‐bridge can enhance the aggregation of the material. Meanwhile, compared with the solution state, both P(gTDPPT) and P(gTDPPSe) exhibited a redshift in their spectra in the thin film, mainly due to further aggregation of the two polymers. Moreover, a distinct 0–0/0–1 vibronic feature was identified in the film spectra, suggesting good planarity and well‐ordered molecular organization and packing for both molecules [27, 56]. Further, the electrochemical properties and energy levels of the two polymers were investigated using cyclic voltammetry (CV). Acetonitrile containing 0.1 m tetrabutylammonium hexafluorophosphate (Bu_4_NPF_6_) served as the electrolyte, and a platinum plate was used as the working electrode. As shown in Figure 2d and Figure S6, P(gTDPPT) and P(gTDPPSe) exhibited distinct reduction and oxidation peaks.

**TABLE 1:** Optical and electrochemical properties of P(gTDPPSe) together with thecontrast polymer P(gTDPPT).

Polymer	*M*_n_ ^a^ [KDa]	PDI	*λ*_max_^solu.^ ^b^ [nm]	*λ*_onset_^film^ ^c^ [nm]	*E*_g_^opt^ ^d^ [eV]	*E*_LUMO_ ^e^ [eV]	*E*_HOMO_ ^e^ [eV]
P(gTDPPSe)	15.4	1.94	830	870	1.43	−3.67	−4.94
P(gTDPPT)	13.1	1.76	804	840	1.48	−3.58	−4.97

^a^ Determined by gel permeation chromatography (GPC) using polystyrene as the standard and hexafluoroisopropanol as the eluent;

^b^ Absorption of polymer chloroform solution (10^−5^ m);

^c^ Absorption of as‐cast film from 10 mg mL^−1^ chloroform solution;

^d^ Calculated from the optical absorption onset of polymer film using the equation *E*_g_^opt^ = 1240/*λ*_onset_^film^ (eV);

^e^ Estimated from *E*_LUMO_ = ‐[*E*_red_^onset^ + 4.80] (eV), *E*_HOMO_ = ‐[*E*_ox_^onset^ + 4.80] eV. *E*_red_^onset^ and *E*_ox_^onset^ are determined by cyclic voltammogram of polymer film measured in 0.1 M tetra(*n*‐butyl)ammonium hexafluorophosphate acetonitrile solution with Fc/Fc^+^ as the external standard.

In addition, based on CV measurements, the *E*_LUMO_/*E*_HOMO_ of P(gTDPPT) were estimated to be −3.58/−4.97 eV, and those of P(gTDPPSe) were −3.67/−4.94 eV (Table 1), which was highly consistent with DFT calculations and UV—vis–NIR characteristics. They all indicated that the presence of the selenophene *π*‐bridge can narrow the material's bandgap. CV showed that the HOMO level (−4.94 eV) and LUMO level (−3.67 eV) of P(gTDPPSe) both fall around the electrochemical window of aqueous electrolytes, indicating that P(gTDPPSe) can be reversibly doped by cations and anions, which explains why P(gTDPPSe) had good ambipolar transport characteristics.

UV–vis‐near‐infrared spectroelectrochemical measurements were conducted in 0.01 m Phosphate‐Buffered Saline (PBS) electrolytes to study the ion doping of the two polymers. As shown in Figure 2e,f, spectral changes were measured by applying different voltages. When gradually increasing negative voltages (0 to −0.9 V, Figure 2e) were applied to the Ag/AgCl electrode, a significant decrease in the intensity of the P(gTDPPSe) ICT absorption peak (around 800 nm) was observed, while the polarization peak at 1050 nm slightly increased, indicating that can achieve effective n‐doping polarization capability. When positive voltages (0 to 0.7 V, Figure 2f) were applied, the ICT absorption peak (around 800 nm) was significantly reduced. These results indicate that P(gTDPPSe) exhibits effective p‐type and n‐type doping. This confirmed that the selenophene *π*‐bridge enables more effective ambipolar doping in P(gTDPPSe), providing a viable route to high‐performance ambipolar OECTs.

**FIGURE 3:**
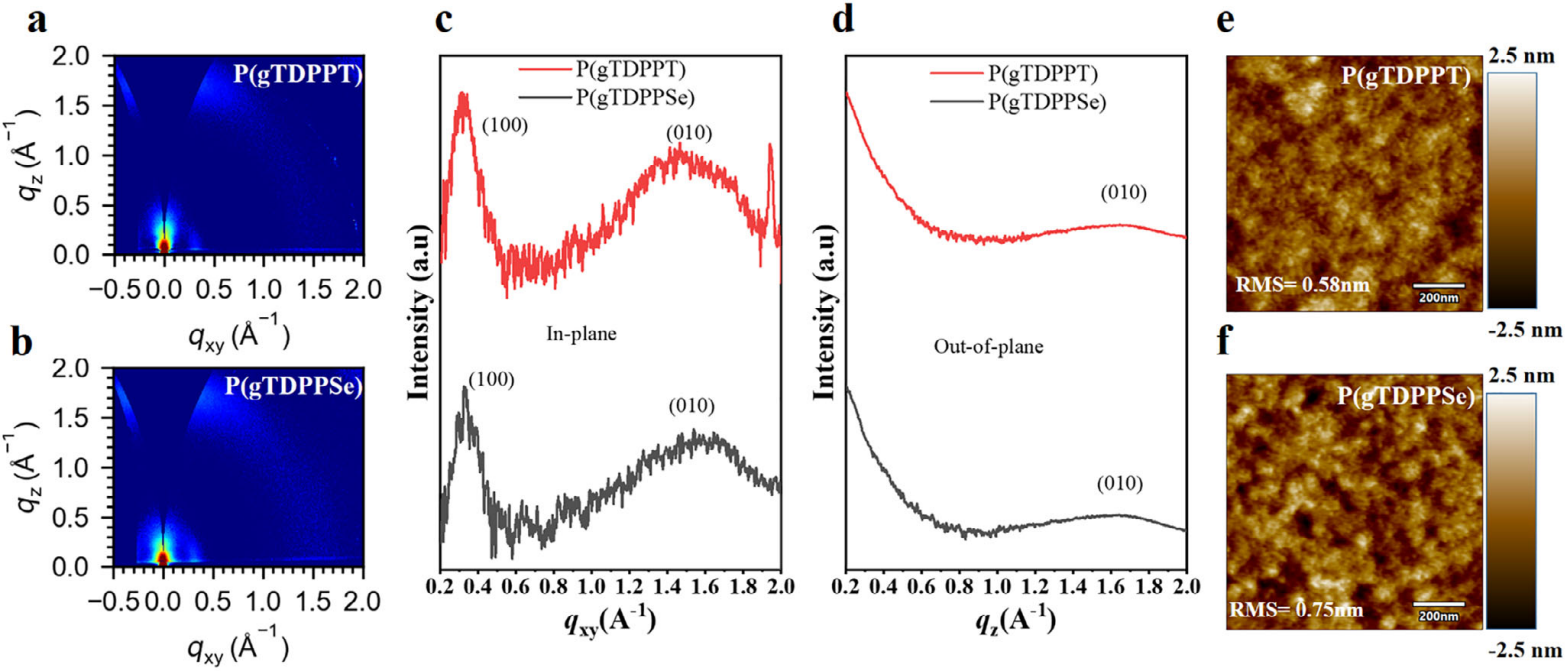
2D‐GIWAXS images of (a) patterned P(gTDPPT) films and (b) patterned P(gTDPPSe) films. The line‐cut profiles in the in‐plane (c) and out‐of‐plane (d) directions extracted from 2D‐GIWAXS patterns. AFM height images (scale bar: 200 nm) of (e) patterned P(gTDPPT) films and (f) patterned P(gTDPPSe) films.

The above measurements confirmed that the selenophene *π*‐bridge satisfies the energy level and ion transport requirements for ambipolar operation. To further elucidate its role in molecular packing and film morphology, we conducted two‐dimensional grazing‐incidence wide‐angle x‐ray scattering (2D‐GIWAXS) and atomic force microscope (AFM) analyses. The 2D‐GIWAXS patterned films (Figure 3a,b) further reveal that both polymers adopt a predominant face‐on orientation with a pronounced diffraction peak corresponding to (010) *π*‐stacking in the out‐of‐plane direction. This orientation is favorable for charge transport between the source and the drain electrodes in vOECTs [30]. And detailed line cutting analysis in Figure 3c,d. Based on the q values of the (100) diffraction peaks, the corresponding lamellar distances of two polymer films were calculated to be 18.47 and 19.53 Å for P(gTDPPSe) and P(gTDPPT). The reduced lamellar distance in P(gTDPPSe) enhances inter‐chain interaction, improving carrier mobility. Based on the out‐of‐plane direction (010) data representation (Table S1), the *π*–*π* stacking distances of the films of P(gTDPPSe) and P(gTDPPT), were 3.89 and 3.93 Å, indicating a significant enhancement of *π*‐stacking interactions within the selenophene‐based polymer. This also suggested that the closer *π*‐stacking distance of P(gTDPPSe) facilitates efficient interchain charge hopping between devices, thereby improving its vOECTs performance. To further reveal the crystal differences, the crystal coherence length (Lc) was analyzed based on the full width at half maximum (FWHM) of the (100) and (010) reflection peaks, using the Scherrer equation (Lc = 2*π*K/FWHM, K = 0.9). The Lc (010) values of P(gTDPPSe) and P(gTDPPT) were similar, being 9.01 and 9.00 Å, respectively. However, the Lc (100) values of P(gTDPPSe) and P(gTDPPT) were 29.32 and 27.97 Å, which indicated more ordered lamellar stacking structures of P(gTDPPSe) compared to P(gTDPPT). Therefore, given that P(gTDPPSe) has reduced interchain *π*‐stacking distance and higher film crystallinity, this polymer also achieved more efficient charge transport in the corresponding vOECTs devices. The surface morphology of spin‐coated polymer films was investigated using AFM (Figure 3e,f). The AFM resultsrevealed root‐mean‐square (RMS) roughness values of 0.75 nm for patterned P(gTDPPSe) and 0.58 nm for patterned P(gTDPPT). Both polymers formed dense films, though sparse network structures with convex particles and visible pinholes were observed. The low roughness indicated that the films of both polymers are relatively ordered, which may be beneficial for charge transport.

**FIGURE 4:**
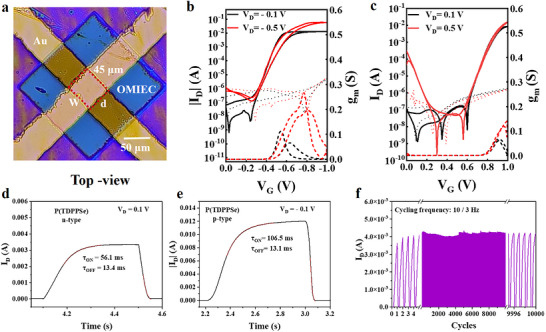
(a) Top‐view optical photograph of vOECTs. (b,c) Ambipolar transfer and transconductance characteristics of patterned P(gTDPPSe)‐based vOECTs in a 0.01 m PBS aqueous electrolyte. (d,e) Ambipolar transient characteristics of patterned P(gTDPPSe)‐based vOECTs in a 0.01 m PBS aqueous electrolyte. (f) Operational stability measurements in 0.01 m PBS aqueous electrolyte for patterned P(gTDPPSe) for n‐type (*V*_G_ = 0.5 to 1.0 V, *V*_D_ = 0.1 V).

Next, vOECTs were introduced to further examine the performances of both polymers [57]. Previous studies have demonstrated that vOECTs exhibit significantly enhanced performance due to improved charge injection efficiency, especially for n‐type materials. The detailed information can be found in Supporting information. Note, a photocurable agent, namely, DtFDA (a redox‐inactive small molecular) is blended with the OMIECs as the channel layer [58, 59]. The top‐view optical photograph of vOECTs was presented in Figure 4a. The output curves of vOECTs with P(gTDPPSe) and P(gTDPPT) were measured in Figure S7. For each kind of vOECTs, at least 8 devices are measured and analyzed (Figure S8), which demonstrated remarkable performance in both p‐type and n‐type modes, achieving *I*_ON_ of 54.9 mA (*V*_DS_ = – 0.5 V and *V*_G_ = – 1.0 V) and 12.9 mA (*V*_DS_ = + 0.5 V and *V*_G_ = + 1.0 V) (Figure S8) and peak *g*_m_ as high as 192.9 and 117.6 mS in Table 2. Despite the ultrasmall channel lengths (L ≈ 60 nm), *I*_ON_/*I*_OFF_ ratios of both devices were ≥10^6^, largely due to the high *I*_ON_ and low *I*_OFF_. The transfer curves of the p‐type and n‐type vOECTs are shown in Figure 4b,c, with the turn‐on voltages (*V*_ON_) being −0.22 and 0.54 V, respectively. Furthermore, we used the P(gTDPPT) as the vOECTs channel to characterize its performance. P(gTDPPT) achieved *I*_ON_ of 45.2 mA (*V*_DS_ = −0.5 V and *V*_G_ = −1.0 V) and peak *g_m_* as 137.6 mS in Figure S9. This also indicated that constructing a selenophene *π*‐bridge by substituting selenium in the *π*‐bridge can provide nearly a 34% increase in p‐type transconductance. This demonstrated that in the synthesis of high‐performance ambipolar OECTs materials, constructing a simple selenophene *π*‐bridge provided a new and straightforward strategy.

**TABLE 2:** Summary of the vOECTs performance parameters of patterned P(gTDPPSe) together with the control polymer patterned P(gTDPPT).

Polymer		*V*_D_ [V]	Device area [µm^2^]	*I*_ON_ [mA]	*V*_ON_ [V]	*g*_m,_ [mS]	*τ*_ON_/*τ*_OFF_ [ms]^a^
P(gTDPPSe)	n	0.5	45 × 45	12.9 ± 1.1	0.54 ± 0.03	113.6 ± 4.0	56.1 ± 2.3 / 13.4 ± 1.6
	p	−0.5	45 × 45	54.9 ± 2.4	−0.22 ± 0.06	174.8 ± 18.1	106.5 ± 4.9 / 13.1 ± 2.1
P(gTDPPT)	p	−0.5	45 × 45	45.2. ± 2.6	−0.22 ± 0.07	137.6 ± 6.1	202.1 ± 8.9 / 19.5 ± 3.6

All the results are based on 8 valid statistics.

^a^ The transient characteristics is measured under *V*_D_ = 0.1 V for n‐type or −0.1 V for p‐type.

We also characterized the ambipolar performance of P(gTDPPSe) by fabricating coplanar OECTs (cOECTs), with the detailed fabrication process provided in the Supporting Information. As expected, P(gTDPPSe) exhibited typical ambipolar transfer characteristics (Figure S10). In p‐type operation (*V*_DS_ = −0.5 V, *V*_G_ = −1.0 V), the maximum drain current (*I*_D_) reached 0.203 mA, with a peak normalized transconductance (g*_m,norm_*) of 28.08 S cm^−1^ (Figure S10). In n‐type operation, the device achieved a maximum *I*_D_ of 0.0115 mA (*V*_DS_ = 0.5 V, *V*_G_ = 1.1 V) and a peak g*_m,norm_* of 1.42 S cm^−1^ (Table S2). From these data, the ambipolar P(gTDPPSe) yielded maximum µC^*^ values of 202.10 F cm^−1^ V^−1^ s^−1^ for p‐type and 12.26 F cm^−1^ V^−1^ s^−1^ for n‐type in cOECTs (Table S2). For comparison, we also characterized the control polymer P(gTDPPT) in cOECT configurations. In p‐type cOECTs, P(gTDPPT) delivered an *I*_D_ of 0.0323 mA (*V*_DS_ = −0.5 V, *V*_G_ = −1.0 V), a g*_m,norm_* of 3.39 S cm^−1^, and a maximum µC^*^ of 133.68 F cm^−1^ V^−1^ s^−1^ (Table S2). These results indicate that the beneficial effects of the selenophene *π*‐bridge are not limited to vOECTs, but also extend to cOECTs.

Then, electrochemical impedance spectroscopy (EIS) was performed to determine the volumetric capacitances (C^*^). As shown in Figure S11, Nyquist plot fitting yielded average C^*^ values of 96.3/76.5 F cm^−3^ (n/p‐type) for patterned P(gTDPPSe) and 42.9 F cm^−3^ (p‐type) for patterned P(gTDPPT). The volumetric capacitance of P(gTDPPSe) was more than twice that of P(gTDPPT) in p‐type, which indicated that P(gTDPPSe) had better ion storage, resulting in higher transconductance performance. This also highlighted the great effectiveness of the selenophene *π*‐bridge strategy in designing high‐performance ambipolar OECTs materials.

To determine the volumetric capacitance of pristine P(gTDPPSe) films, we performed EIS measurements on the pure films, yielding average C^*^ values of 140.3 F cm^−3^ (n‐type) and 109.4 F cm^−3^ (p‐type). For comparison, the pristine P(gTDPPT) film gave a p‐type C^*^ of 48.6 F cm^−3^. After cross‐linking and patterning, the volumetric capacitance of P(gTDPPSe) films decreased (31% for n‐type, 30% for p‐type), yielding C^*^ values of 96.3 F cm^−3^ (n‐type) and 76.5 F cm^−3^ (p‐type) (Figure S12). In return, the cross‐linked films offer improvements in three key points: (i) enabling precise patterning to define the vertical channel geometry; (ii) providing sufficient mechanical robustness for vertical device fabrication; and (iii) ensuring reproducible device performance across multiple batches. We therefore consider this trade‐off—sacrificing some ion storage capacity for enhanced physical stability and patterning precision—to be well justified.

We used electrochemical quartz crystal microbalance (EQCM) to study the effect of the selenophene *π*‐bridge on film swelling behavior. By quantifying the relative mass change under applied bias, we evaluated the differences in film swelling between the two materials. When a + 0.8 V bias (vs. Ag/AgCl) was applied, both films exhibited a mass increase, indicating good anion doping capability for both. Notably, the active mass of P(gTDPPSe) increased by 2.8% upon doping, while P(gTDPPT) increased by only 1.8% (Figure S13). These results demonstrate that, by enhancing film swelling behavior and ion permeability, the selenophene bridge in P(gTDPPSe) contributes to its superior anion doping capability. Additionally, from the EQCM test graphs of P(gTDPPSe) under two opposite voltages (Figure S13), we found that n‐type can undergo effective doping and dedoping, whereas the p‐type exhibits difficult dedoping after doping which may be another important reason for the poor stability of p‐type.

We evaluated the transient response of the ambipolar vOECTs by comparing their response times. In n‐type performance, the switch‐on time (*τ*_ON_) and switch‐off time (*τ*_OFF_) of P(gTDPPSe) were 56.1 and 13.4 ms (at *V*_D_ = 0.1 V) (Figure 4d). While in p‐type performance, the *τ*_ON_ and *τ*_OFF_ of P(gTDPPSe) were 106.5 ms and 13.1 ms (at *V*_D_ = − 0.1 V) (Figure 4e). Meanwhile, we also characterized the response times of P(gTDPPT) in p‐type performance, where the *τ*_ON_ and *τ*_OFF_ of P(gTDPPT) were 202.1 and 19.5 ms (at *V*_D_ = −0.1 V) (Figure S9b). This showed that P(gTDPPSe) had a shorter response time in p‐type performance. Later, we conducted stability tests on the two materials, as shown in Figure 4f and Figure S14. Under n‐type conditions, P(gTDPPSe) still maintained 96% of its current after 10 000 cycles. However, P(gTDPPSe) only maintained 81% of its current after 100 cycles in the p‐type, and P(gTDPPT) retained only 70% after 80 cycles.

To investigate the origin of the rapid p‐type degradation, we performed five consecutive CV cycles in 0.01 m PBS solution under ambient conditions. As shown in Figure S15, the oxidation peak current decreased significantly over the five cycles, while the reduction peak current remained stable. This indicates that the instability is specifically associated with the oxidation (p‐type) process. In addition, we performed AFM characterization before and after 100 p‐type cycles and 1000 n‐type cycles. The AFM images (Figure S16) show that the surface roughness significantly increased after the p‐type cycles (RMS from 0.75 to 2.96 nm), whereas only a slight increase was observed after n‐type doping (RMS from 0.75 to 0.93 nm). This indicates that p‐type doping causes irreversible morphological changes in the film, which may be attributed to the inability of p‐type doping to be effectively dedoped (Figure S13). In summary, the poor p‐type stability of P(gTDPPSe) is attributed to two factors: (i) the instability of oxidative intermediates (radical cations) against water/oxygen electrochemical side reactions, and (ii) irreversible morphological changes caused by the inability to effectively dedope after p‐type doping.

**FIGURE 5:**
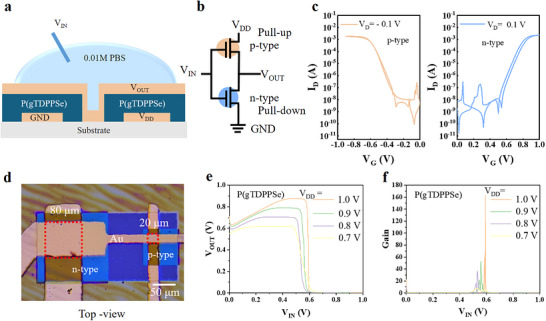
(a) Schematic diagram of OECT‐based complementary logic circuit with P(gTDPPSe) as single‐component channel material, and (b) corresponding circuit configuration. (c) Transfer curves of the OECTs in p‐type and n‐type operational modes. (d) Top‐view optical photograph of the complementary inverter of patterned P(gTDPPSe). (e) Typical voltage transfer characteristics of the inverter at supply voltages. (f) Gains are calculated from the slope of the voltage transfer characteristics.

By utilizing OECTs based on P(gTDPPSe) as a single‐component channel material, we successfully fabricated single‐element complementary logic inverters. These inverters consisted of two identical OECTs (pull‐up and pull‐down) using patterned P(gTDPPSe) as the channel material. The architecture design and corresponding circuit diagram were shown in Figure 5a,b.The input voltage (*V_IN_*) is connected to the Ag/AgCl gate electrodes of both OECTs, while the output voltage (*V*_OUT_) is monitored externally in series between the transistors. Figure 5c showed that the voltage‐current transfer curve of the pull‐up OECTs is fairly symmetric with that of the pull‐down OECTs. The top‐view optical photograph of the complementary inverter of P(gTDPPSe) showed in Figure 5d. The voltage transfer characteristics (*VTC*) were evaluated at varying supply voltages of 0.7, 0.8, 0.9, and 1.0 V by scanning the *V*_IN_ from 0.7 to 1.0 V, with the resultant *VTC* and voltage‐gain curves depicted in Figure 5e,f. By applying positive bias voltage, we measured the voltage transfer curves and values of the inverters. The P(gTDPPSe)‐based inverter obtained a much higher gain value (163 V/V), which is an outstanding performance reported to date among single‐component OECT‐based complementary inverters (Table S3).

These results demonstrated that the selenophene *π*‐bridge design not only enables high‐performance OECTs inverters, but also provides a promising platform for complementary logic circuits targeting complex signal processing and bioelectronic interfacing.

## 3 Conclusion

In summary, we have successfully synthesized two DPP‐based polymers, P(gTDPPSe) and P(gTDPPT), which are copolymerized with selenophene and thiophene, respectively. Through a combination of DFT calculations, optical spectroscopy, and EIS characterization, we conducted a comprehensive analysis. We demonstrate that, compared with P(gTDPPT) copolymerized with thiophene, P(gTDPPSe) via incorporating a selenophene π‐bridge, exhibited favorable energy alignment, a narrow bandgap, and balanced ambipolar **transport**, together with high C^*^. Meanwhile, we also found that P(gTDPPSe) had a smaller interchain π‐π stacking distance and higher film crystallinity by 2D‐GIWAXS, demonstrating that the selenophene π‐bridge enabled more efficient charge transport. All the above measurements have proven that P(gTDPPSe) synthesized with the selenophene π‐bridge meets all the requirements of an ambipolar OECT material. Then, vOECTs of P(gTDPPSe) were fabricated to characterize the ambipolar performance, which also demonstrated remarkable performance in both p‐type and n‐type modes, achieving *I_ON_* of 57.3 mA and 14.0 mA and peak *g_m_* as high as 192.9 and 117.6 mS, respectively, due to the balanced ambipolar performance of P(gTDPPSe). Subsequently, we fabricated inverters achieving a maximum gain of 163 V/V, demonstrating that high transconductance and balanced p–n characteristics are essential for high performance. This work establishes selenophene π‐bridge engineering as an effective strategy to develop balanced ambipolar OMIECs for complementary logic circuits.

## Conflicts of Interest

The authors declare no conflict of interest.

## Supporting information




**Supporting File**: advs76016‐sup‐0001‐SuppMat.docx.

## Data Availability

The data that support the findings of this study are available from the corresponding author upon reasonable request.

## References

[1] L. M. M. Ferro , L. Merces , D. H. S. de Camargo , and C. C. Bof Bufon , “Ultrahigh‐Gain Organic Electrochemical Transistor Chemosensors Based on Self‐Curled Nanomembranes,” Advanced Materials 33 (2021): 2101518, 10.1002/adma.202101518. 34061409

[2] P. Gkoupidenis , Y. Zhang , H. Kleemann , et al., “Organic Mixed Conductors for Bioinspired Electronics,” Nature Reviews Materials 9 (2024): 134–149, 10.1038/s41578-023-00622-5.

[3] R. He , A. Lv , X. Jiang , et al., “Organic Electrochemical Transistor Based on Hydrophobic Polymer Tuned by Ionic Gels,” Angewandte Chemie International Edition 62 (2023): 202304549, 10.1002/anie.202304549. 37439325

[4] L. Huang , Z. Wang , J. Chen , et al., “Porous Semiconducting Polymers Enable High‐Performance Electrochemical Transistors,” Advanced Materials 33 (2021): 2007041, 10.1002/adma.202007041. 33655643

[5] R. Liu , X. Zhu , J. Duan , et al., “Versatile Neuromorphic Modulation and Biosensing based on N‐type Small‐molecule Organic Mixed Ionic‐Electronic Conductors,” Angewandte Chemie International Edition 63 (2024): 202315537, 10.1002/anie.202315537. 38081781

[6] M. Ma , L. Zhang , M. Huang , et al., “Regiochemistry and Side‐Chain Engineering Enable Efficient N‐Type Mixed Conducting Polymers,” Angewandte Chemie International Edition 64 (2025): 202424820. 10.1002/anie.202424820 40087895

[7] R. B. Rashid , X. Ji , and J. Rivnay , “Organic Electrochemical Transistors in Bioelectronic Circuits,” Biosensors and Bioelectronics 190 (2021): 113461, 10.1016/j.bios.2021.113461. 34197997

[8] J. Rivnay , S. Inal , A. Salleo , R. M. Owens , M. Berggren , and G. G. Malliaras , “Organic Electrochemical Transistors,” Nature Reviews Materials 3 (2018): 17086, 10.1038/natrevmats.2017.86.

[9] H. Sun , M. Vagin , S. Wang , et al., “Complementary Logic Circuits Based on High‐Performance n‐Type Organic Electrochemical Transistors,” Advanced Materials 30 (2018): 1704916, 10.1002/adma.201704916. 29318706

[10] L. Tang , X. Zheng , M. Sun , et al., “Photopatternable Gel Electrolytes for Stretchable Solid‐State Organic Electrochemical Transistors,” Science China Materials 68 (2025): 3212–3218, 10.1007/s40843-025-3429-x.

[11] A. Giovannitti , D.‐T. Sbircea , S. Inal , et al., “Controlling the Mode of Operation of Organic Transistors Through Side‐Chain Engineering,” Proceedings of the National Academy of Sciences 113 (2016): 12017–12022, 10.1073/pnas.1608780113. PMC5087003 27790983

[12] L. Travaglini , K. Fidanovski , and D. Mawad , “Logic Circuits Featuring Organic Electrochemical Transistors: What is the Logic Behind OECTs in Logic?,” Advanced Science 12 (2025): 14448, 10.1002/advs.202514448. PMC12697826 41168977

[13] Y. Wang , G. Zhu , E. Zeglio , et al., “n‐Type Organic Electrochemical Transistors with High Transconductance and Stability,” Chemistry of Materials 35 (2023): 405–415.

[14] A. Savva , R. Hallani , C. Cendra , et al., “Balancing Ionic and Electronic Conduction for High‐Performance Organic Electrochemical Transistors,” Advanced Functional Materials 30 (2020): 1907657, 10.1002/adfm.201907657.

[15] G.‐Y. Ge , J. Xu , X. Wang , et al., “On‐Site Biosignal Amplification Using a Single High‐Spin Conjugated Polymer,” Nature Communications 16 (2025): 396, 10.1038/s41467-024-55369-6. PMC11700166 39755691

[16] J. Li , Z. Li , J. Huang , et al., “High‐Performance Ambipolar Organic Electrochemical Transistors Based on Diketopyrrolopyrrole‐Dialkoxybithiazole Conjugated Polymers for Single‐component Inverters,” Advanced Science 13 (2026): 20003, 10.1002/advs.202520003. PMC13042758 41560630

[17] G. Qi , M. Wang , S. Wang , et al., “High‐Performance, Single‐Component Ambipolar Organic Electrochemical Transistors With Balanced n/p ‐Type Properties for Inverter and Biosensor Applications,” Advanced Functional Materials 35 (2025): 2413112, 10.1002/adfm.202413112.

[18] R. B. Rashid , W. Du , S. Griggs , I. P. Maria , I. McCulloch , and J. Rivnay , “Ambipolar Inverters Based on Cofacial Vertical Organic Electrochemical Transistor Pairs for Biosignal Amplification,” Science Advances 7 (2021): abh1055, 10.1126/sciadv.abh1055. PMC8442873 34516877

[19] E. Stein , O. Nahor , M. Stolov , et al., “Ambipolar Blend‐Based Organic Electrochemical Transistors and Inverters,” Nature Communications 13 (2022): 5548, 10.1038/s41467-022-33264-2. PMC9500051 36137998

[20] Y. Lei , P. Li , Y. Zheng , and T. Lei , “Materials Design and Applications of n‐Type and Ambipolar Organic Electrochemical Transistors,” Materials Chemistry Frontiers 8 (2024): 133–158, 10.1039/D3QM00828B.

[21] J. Yang , Z. Zhao , H. Geng , et al., “Isoindigo‐Based Polymers With Small Effective Masses for High‐Mobility Ambipolar Field‐Effect Transistors,” Advanced Materials 29 (2017): 1702115, 10.1002/adma.201702115. 28736833

[22] Y. Ren , X. Yang , L. Zhou , J.‐Y. Mao , S.‐T. Han , and Y. Zhou , “Recent Advances in Ambipolar Transistors for Functional Applications,” Advanced Functional Materials 29 (2019): 1902105, 10.1002/adfm.201902105.

[23] C. G. Tang , M. C. Y. Ang , K.‐K. Choo , et al., “Doped Polymer Semiconductors With Ultrahigh and Ultralow Work Functions for Ohmic Contacts,” Nature 539 (2016): 536–540, 10.1038/nature20133. 27882976

[24] K. Zhou , H. Dong , H.‐L. Zhang , and W. Hu , “High Performance n‐type and Ambipolar Small Organic Semiconductors for Organic Thin Film Transistors,” Physical Chemistry Chemical Physics 16 (2014): 22448–22457, 10.1039/C4CP01700E. 24993863

[25] N. A. Kukhta , A. Marks , and C. K. Luscombe , “Molecular Design Strategies Toward Improvement of Charge Injection and Ionic Conduction in Organic Mixed Ionic–Electronic Conductors for Organic Electrochemical Transistors,” Chemical Reviews 122 (2022): 4325–4355, 10.1021/acs.chemrev.1c00266. 34902244 PMC8874907

[26] J. J. Samuel , A. Garudapalli , A. A. Mohapatra , C. Gangadharappa , S. Patil , and N. P. B. Aetukuri , “Single‐Component CMOS‐Like Logic using Diketopyrrolopyrrole‐Based Ambipolar Organic Electrochemical Transistors,” Advanced Functional Materials 31 (2021): 2102903, 10.1002/adfm.202102903.

[27] L. Lan , Y. Wang , X. Zhu , I. McCulloch , and W. Yue , “Ultrathin‐Film Small Molecule Mixed Conductors Exhibiting Ion‐Tunable Ambipolarity for High‐Performance Organic Electrochemical Transistors and Multivalued Logic Inverters,” Advanced Materials 37 (2025): 2501041, 10.1002/adma.202501041. 40589189

[28] A. Giovannitti , C. B. Nielsen , D.‐T. Sbircea , et al., “N‐Type Organic Electrochemical Transistors With Stability in Water,” Nature Communications 7 (2016): 13066, 10.1038/ncomms13066. PMC5059848 27713414

[29] Y. Wang , J. Tan , H. Hou , et al., “High Performing Ambipolar Organic Electrochemical Transistors and Solid‐State Inverters Enabled by Hydrophilic/Hydrophobic Side Chains Integration,” Advanced Materials 38 (2026): 15186, 10.1002/adma.202515186. 41340327

[30] Y. Sun , Y. Lan , M. Li , et al., “Indacenodithiophene‐Based Single‐Component Ambipolar Polymer for High‐Performance Vertical Organic Electrochemical Transistors and Inverters,” Aggregate 5 (2024): 577, 10.1002/agt2.577.

[31] M. Wang , Z. Yan , Q. Yun , et al., “Self‐Powered Broadband Underwater Optical Communication Enabled by Enhanced Donor‐Acceptor Molecular Junction in *π*‐Conjugated Covalent Organic Framework,” Chemical Engineering Journal 524 (2025): 169368, 10.1016/j.cej.2025.169368.

[32] Y. Gao , Y. Ke , T. Wang , et al., “An n‐Type Conjugated Polymer With Low Crystallinity for High‐Performance Organic Thermoelectrics,” Angewandte Chemie International Edition 63 (2024): 202402642, 10.1002/anie.202402642. 38453641

[33] I.‐Y. Jo , D. Jeong , Y. Moon , et al., “High‐Performance Organic Electrochemical Transistors Achieved by Optimizing Structural and Energetic Ordering of Diketopyrrolopyrrole‐Based Polymers,” Advanced Materials 36 (2024): 2307402, 10.1002/adma.202307402. 37989225

[34] M. Moser , A. Savva , K. Thorley , et al., “Polaron Delocalization in Donor–Acceptor Polymers and its Impact on Organic Electrochemical Transistor Performance,” Angewandte Chemie International Edition 60 (2021): 7777–7785, 10.1002/anie.202014078. 33259685

[35] Y. Wang , R. Wang , F. Li , et al., “Highly Sensitive All‐Polymer Short‐Wavelength Infrared Photodetectors for Complementary Metal Oxide Semiconductor Integrated Imaging Enabled by Dual‐Acceptor n‐Type Polymers,” Angewandte Chemie International Edition 65 (2026): 25467, 10.1002/anie.202525467. 41703757

[36] X. Wu , Q. Liu , A. Surendran , S. E. Bottle , P. Sonar , and W. L. Leong , “Enhancing the Electrochemical Doping Efficiency in Diketopyrrolopyrrole‐Based Polymer for Organic Electrochemical Transistors,” Advanced Electronic Materials 7 (2021): 2000701, 10.1002/aelm.202000701.

[37] J. Liu , X. Wang , H. Zhu , Y. Li , B. Lv , and W. Qiao , “π‐Bridge‐Engineered Naphthalenediimide Copolymers for all‐Polymer Photodetectors With High Specific Detectivity,” Polymer 335 (2025): 128852, 10.1016/j.polymer.2025.128852.

[38] A. Patra and M. Bendikov , “Polyselenophenes,” Journal of Materials Chemistry 20 (2010): 422–433, 10.1039/B908983G.

[39] X. Zhong , H. Chen , M. Wang , et al., “Synergistic Effect of Chlorination and Selenophene: Achieving Elevated Solar Conversion in Highly Aggregated Systems,” Macromolecules 52 (2019): 2393–2401, 10.1021/acs.macromol.8b02445.

[40] H.‐Y. Chen , S.‐C. Yeh , C.‐T. Chen , and C.‐T. Chen , “Comparison of Thiophene‐ And Selenophene‐Bridged Donor–Acceptor Low Band‐Gap Copolymers Used in Bulk‐Heterojunction Organic Photovoltaics,” Journal of Materials Chemistry 22 (2012): 21549–21559, 10.1039/c2jm33735e.

[41] M. Li , W. Feng , Y. Lan , et al., “Effects of Selenium Incorporation on the Performance of Polythiophene Based Organic Electrochemical Transistors,” Journal of Materials Chemistry C 12 (2024): 7935–7942, 10.1039/D4TC01226G.

[42] A. V. Marsh and M. Heeney , “Conjugated Polymers Based on Selenophene Building Blocks,” Polymer Journal 55 (2023): 375–385, 10.1038/s41428-022-00731-y.

[43] R. S. Ashraf , I. Meager , M. Nikolka , et al., “Chalcogenophene Comonomer Comparison in Small Band Gap Diketopyrrolopyrrole‐Based Conjugated Polymers for High‐Performing Field‐Effect Transistors and Organic Solar Cells,” Journal of the American Chemical Society 137 (2015): 1314–1321, 10.1021/ja511984q. 25547347

[44] I. Kang , T. K. An , J. Hong , et al., “Effect of Selenophene in a DPP Copolymer Incorporating a Vinyl Group for High‐Performance Organic Field‐Effect Transistors,” Advanced Materials 25 (2013): 524–528, 10.1002/adma.201202867. 23125035

[45] J. Kim , X. Ren , Y. Zhang , et al., “Efficient N‐Type Organic Electrochemical Transistors and Field‐Effect Transistors Based on PNDI‐Copolymers Bearing Fluorinated Selenophene‐Vinylene‐Selenophenes,” Advanced Science 10 (2023): 2303837, 10.1002/advs.202303837. 37551064 PMC10582458

[46] R. L. Uy , L. Yan , W. Li , and W. You , “Tuning Fluorinated Benzotriazole Polymers Through Alkylthio Substitution and Selenophene Incorporation for Bulk Heterojunction Solar Cells,” Macromolecules 47 (2014): 2289–2295, 10.1021/ma5001095.

[47] G. Liu , M. Zhang , J. Lv , et al., “High Iontronic Performance in Organic Electrochemical Transistors Enabled by Intramolecular Noncovalent Interactions,” Advanced Materials 38 (2026): 08541, 10.1002/adma.202508541. 41055297

[48] W. Wu , K. Feng , Y. Wang , et al., “Selenophene Substitution Enabled High‐Performance n‐Type Polymeric Mixed Ionic‐Electronic Conductors for Organic Electrochemical Transistors and Glucose Sensors,” Advanced Materials 36 (2024): 2310503, 10.1002/adma.202310503. 37961011

[49] J. Li , M. Liu , K. Yang , et al., “Selenium Substitution in Bithiophene Imide Polymer Semiconductors Enables High‐Performance n ‐Type Organic Thermoelectric,” Advanced Functional Materials 33 (2023): 2213911, 10.1002/adfm.202213911.

[50] H. Huang , L. Yang , A. Facchetti , and T. J. Marks , “Organic and Polymeric Semiconductors Enhanced by Noncovalent Conformational Locks,” Chemical Reviews 117 (2017): 10291–10318, 10.1021/acs.chemrev.7b00084. 28671815

[51] S. Cong , J. Chen , M. Xie , et al., “Single Ambipolar OECT–Based Inverter With Volatility and Nonvolatility on Demand,” Science Advances 10 (2024): adq9405, 10.1126/sciadv.adq9405. PMC11463256 39383214

[52] X. Chen , Z. Zhang , Z. Ding , J. Liu , and L. Wang , “Diketopyrrolopyrrole‐based Conjugated Polymers Bearing Branched Oligo(Ethylene Glycol) Side Chains for Photovoltaic Devices,” Angewandte Chemie International Edition 55 (2016): 10376–10380, 10.1002/anie.201602775. 27258171

[53] P. Li , J. Shi , Y. Lei , Z. Huang , and T. Lei , “Switching p‐Type to High‐Performance n‐Type Organic Electrochemical Transistors via Doped State Engineering,” Nature Communications 13 (2022): 5970, 10.1038/s41467-022-33553-w. PMC9551099 36216813

[54] K. Feng , W. Shan , S. Ma , et al., “Fused Bithiophene Imide Dimer‐Based n‐Type Polymers for High‐Performance Organic Electrochemical Transistors,” Angewandte Chemie International Edition 60 (2021): 24198–24205, 10.1002/anie.202109281. 34467624

[55] P. Chao , M. Guo , Y. Zhu , et al., “Enhanced Photovoltaic Performance by Synergistic Effect of Chlorination and Selenophene π‐Bridge,” Macromolecules 53 (2020): 2893–2901, 10.1021/acs.macromol.0c00405.

[56] Y. Zhou , C. Miao , Z. Su , et al., “Varied π‐Conjugated Extension Directions in B←N Coordinated Benzodipyrrolidone (BDP) Polymer Semiconductors: Effects on n‐Type Organic Field‐Effect Transistors (OFETs),” ACS Materials Letters 6 (2024): 5093–5102, 10.1021/acsmaterialslett.4c01654.

[57] W. Huang , J. Chen , Y. Yao , et al., “Vertical Organic Electrochemical Transistors for Complementary Circuits,” Nature 613 (2023): 496–502, 10.1038/s41586-022-05592-2. 36653571 PMC9849123

[58] Y. Lai , J. Cheng , M. Xie , et al., “Precisely Patterned Channels in a Vertical Organic Electrochemical Transistor With a Diazirine Photo‐Crosslinker,” Angewandte Chemie International Edition 63 (2024): 202401773, 10.1002/anie.202401773. 38429971

[59] Y.‐Q. Zheng , Y. Liu , D. Zhong , et al., “Monolithic Optical Microlithography of High‐Density Elastic Circuits,” Science 373 (2021): 88–94, 10.1126/science.abh3551. 34210882

