# Supplementary material for: Selenophene π‐Bridge Enables Balanced Ambipolar Transport in High‐Performance Organic Electrochemical Transistors
Source: Adv Sci (Weinh). 2026 Jun 9:e76016. Online ahead of print. doi: 10.1002/advs.76016 (PMC13336496; doi:10.1002/advs.76016)
Supplement: Supplementary file 1 — Supporting File: advs76016‐sup‐0001‐SuppMat.docx. [file ADVS-9999-e76016-s001.docx]

# Supporting Information

**Selenophene π‑Bridge Enables Balanced Ambipolar Transport in High‑Performance Organic Electrochemical Transistors**

*Guichuan Zhu, ^[a]^ Yueping Lai, ^[a]^ Jiaxing Pu, ^[a]^ Jianhua Chen, ^[b]^ Liang-Wen Feng ^[a]^**

[a] G. Zhu, Y. Lai, J. Pu , Prof. L.-W. Feng
Key Laboratory of Green Chemistry & Technology, Ministry of Education, College of Chemistry, Sichuan University, Chengdu, 610065, China
E-mail: [liangwenfeng@scu.edu.cn](mailto:liangwenfeng@scu.edu.cn)

[b] Prof. J. Chen
Department of Chemical Science and Technology, Yunnan University, Kunming, 650091, China.

**Table of Contents**

[**Supporting Information 1**](#_Toc229330567)

[**1. Experimental and Material Characterization. 3**](#_Toc229330568)

[**1.1. Materials and Synthesis. 3**](#_Toc229330569)

[**1.2 Instruments and Measurement 4**](#_Toc229330570)

[**2. Supplementary Figures and Tables 7**](#_Toc229330571)

[**3. Supplementary References. 20**](#_Toc229330572)

1. Experimental and Material Characterization.

1.1. Materials and Synthesis.

All reagents and chemicals were purchased from commercial sources and were used without further purification. 3,6-Di(2-thienyl)-2,5-dihydropyrrolo[3,4-c]pyrrole-1,4-dione (98%), 2,5-Bis(trimethylstannyl)thiophene (98%) and tri(o-tolyl)phosphine (98%) were purchased from Adamas. 2,5-Selenophenediylbis(trimethylstannane) (98%) was purchased from Derthon. Tris(dibenzylideneacetone)-dipalladium (98%) was purchased from Sigma-Aldrich.

The monomer **g-TDPP** was prepared according to the literature.^[1]^ The synthetic routes to the monomer g-TDPP-Br and two polymers are depicted in **Scheme S1**, respectively. The polymers were purified by Soxhlet extraction and characterized by ^1^H NMR.

**Scheme S1**

**
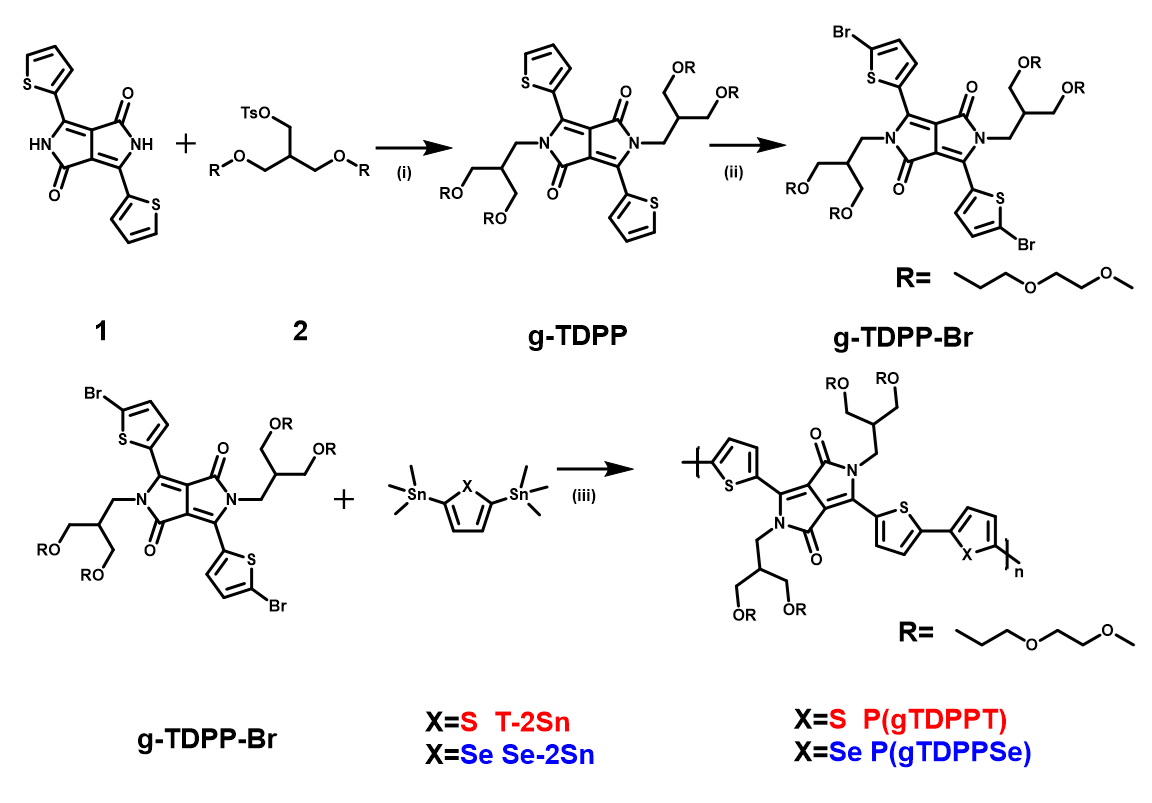
**

Reagents and conditions: (i) K_2_CO_3_, TBAB, DMF, 120 °C, 10 h; (ii) NBS, DCM, 0 °C, 10 h; (iii) Pd_2_(dba)_3_, P(o-tolyl)_3_, Toluene/DMF (v/v = 1/1), 130 °C, 48 or 10 h.

**Synthesis of Compound g-TDPP-Br**

To the solution of g-TDPP (5.15 g, 5.82 mmol) in dichloromethane (100.0 mL), N-Bromosuccinimide (2.18 g, 12.23 mmol) was added at 0 ºC in dark. The mixture was stirred at room temperature for 10 h. Then, the reaction mixture was poured into water and extracted with dichloromethane. The organic phase were washed with water for three times, dried over anhydrous Na_2_SO_4_, filtered and concentrated. The crude product was purified by silica gel chromatography and recrystallization in ethanol to give compound g-TDPP-Br as a dark red crystal (4.25 g, 53.5%). ^1^H NMR (600 MHz, CDCl_3_) δ (ppm) 8.55-8.54 (d, *J* = 6.0 Hz, 2H), 7.23-7.22 (d, *J* = 6.0 Hz, 2H), 4.14-4.12 (d, *J* = 12.0 Hz, 4H), 3.83-3.48 (m, 42H), 3.36 (s, 12H), 2.43 (m, 2H). ^13^C NMR (150.9 MHz, CDCl_3_) δ (ppm) 161.41, 138.97, 134.86, 131.61, 131.40, 119.41, 107.88, 71.97, 70.76, 70.60, 70.53, 70.18, 59.06, 41.79, 40.26.

**Synthesis of Compound P(gTDPPT)**

A mixture of monomer g-TDPP-Br (365.0 mg, 0.35 mmol), 2,5-bis(trimethylstannyl)thiophene 143.5 mg, 0.35 mmol), Pd_2_(dba)_3_ (31.0 mg), P(o-tolyl)_3_ (85.0 mg), degassed PhCl (5.0 mL) and DMF (5 mL) was vigorously stirred at 130 ºC under argon atmosphere and stirred at 130 ºC for 48 h. After cooled down, the resulting mixture was poured into n-hexane (200.0 mL) and the precipitate was collected by filtration. The crude polymer was washed in a Soxhlet apparatus with methanol, acetone, n-hexane and chloroform in sequential. The chloroform fraction was concentrated and poured into n-hexane. The polymer fiber was recovered by filtration and dried in vacuum overnight. Yield: (282.0 mg, 83.5%). ^1^H NMR (600 MHz, CDCl_3_) δ (ppm) 9.34-8.46 (br) 7.33-6.49 (br), 4.08-3.2 (br), 2.63-2.24 (br).

**Synthesis of Compound P(gTDPPSe)**

A mixture of monomer g-TDPP-Br (365.0 mg, 0.35 mmol), 2,5-bis(trimethylstannyl)thiophene 160.0 mg, 0.35 mmol), Pd_2_(dba)_3_ (24.0 mg), P(o-tolyl)_3_ (42.6 mg), degassed PhCl (5.0 mL) and DMF (5 mL) was vigorously stirred at 130 ºC under argon atmosphere and stirred at 130 ºC for 10 h. After cooled down, the resulting mixture was poured into n-hexane (200.0 mL) and the precipitate was collected by filtration. The crude polymer was washed in a Soxhlet apparatus with methanol, acetone, n-hexane and chloroform in sequential. The chloroform fraction was concentrated and poured into n-hexane. The polymer fiber was recovered by filtration and dried in vacuum overnight. Yield: (326.0 mg, 92.0%). ^1^H NMR (600 MHz, CDCl_3_) δ (ppm) 9.27-8.57 (br), 7.34-6.60 (br), 2.66-2.28 (br).

1.2 Instruments and Measurement

**Nuclear Magnetic Resonance (NMR):** ^1^H NMR 600 MHz and ^13^C NMR 150.9 MHz were collected with a Bruker ASCENDTM spectrometer in CDCl_3_.

**Gel Permeation Chromatography (GPC):** Using Hexafluoroisopropanol as the eluent and polystyrene as the standard sample, the molecular weights of the P(gTDPPT) and P(gTDPPSe) were measured on Agilent 1260 at room temperature.

**Thermogravimetric Analysis (TGA):** Thermogravimetric analysis was conducted under nitrogen atmosphere at a heating rate of 10 ºC /min on a TSA 449 F3.

**Differential Scanning Calorimetry (DSC):** Differential scanning calorimetry was carried out on a DSC 3500 at a ±10 ^o^C / min heating/cooling rate at a nitrogen flow.

**UV-vis-NIR Absorption Spectroscopy:** UV-vis-NIR absorption spectroscopy was recorded on Shimadzu UNICO UV-4802 UV-Vis spectrophotometer. Solutions of the polymers were prepared in chloroform (10^-5^ M) and thin films of the solutions (polymer: DtFDA: Cell= 20:1:0.1) were prepared by spin coating 10 mg mL^-1^ chloroform solutions at a speed of 3000 rmp on quartz substrates.

**Spectroelectrochemical Measurements:** Using UNICO UV-4802 UV-Vis spectrophotometer to test the absorption of P(gTDPPT) and P(gTDPPSe) in chloroform solution and neat films. For in-situ UV-vis-NIR measurements, the neat films were prepared by spin-coating a chloroform solution (polymer : DtFDA : Cell = 20:1:0.1) with a concentration of 10 mg/mL on ITO at a speed of 3000 rmp. The annealed ITO with polymer was placed in 0.01 M PBS aqueous solution with a step of 0.1 V to apply a voltage from 0 to 0.7 V (p-type) and from 0 to -0.9 V (n-type), respectively.

**Cyclic Voltammetry (CV):** Cyclic voltammetry (CV) measurement was probed on a Metrohm, µstat-i400. Electrochemical workstation was in an acetonitrile solution of tetra-n-butylammonium hexafluoro-phosphate (Bu_4_NPF_6_) (0.1 M) with a scan rate of 100 mV s^-1^. A conventional three-electrode cell with a platinum plate working electrode, platinum wire counter-electrode, and Ag/AgCl reference electrode was used. Ferrocene/Ferrocenium (Fc/Fc^+^) used as the internal standard. The molecular LUMO and HOMO energy levels were calculated by the following formula:^[2-3]^

HOMO=-[*E*_ox_-*E*(Fc/Fc^+^) + 4.8] eV

LUMO=-[*E*_red_-*E*(Fc/Fc^+^) + 4.8] eV

Where *E*_ox_ and *E_r_*_ed_ are the initial oxidation/reduction potentials of polymers in 0.1 M anhydrous acetonitrile solution of Bu_4_NPF_6_, *E*(Fc/Fc^+^) is the half-wave potential of the Fc/Fc^+^ redox couple. The CV data with variable scan rate were analyzed by Randles-SěvčíK equation.

**Density Functional Theory (DFT) Calculations:** The theoretical calculations were performed via the Gaussian 16 suite of programs. The structures of the studied molecules (denoted by P(gTDPPT) and P(gTDPPSe)) were fully optimized at the B3LYP-D3BJ/def2-SVP level of theory. The vibrational frequencies of the optimized structures were carried out at the same level. The structures were characterized as a local energy minimum on the potential energy surface by verifying that all the vibrational frequencies were real. The molecular orbital levels of studied compounds were investigated at the B3LYP-D3BJ/def2-TZVP level of theory., including the highest occupied molecular orbital (HOMO) and the lowest unoccupied molecular orbital (LUMO). The Visual Molecular Dynamics (VMD) program was used to plot the color-filled iso-surface graphs to visualize the molecular orbitals.

The cationic (+1) and anionic (-1) molecular systems were optimized at the B3LYP-D3(BJ)/def2-SVP level. Their spin density calculations were then performed at the B3LYP-D3(BJ)/def2-TZVP level, and the corresponding isosurfaces were visualized using the VMD software.

**Electrochemical Impedance Spectroscopy (EIS)**: EIS measurements were conducted to obtain the volumetric capacitance (C*) in 0.01 M PBS aqueous solution with a three-electrode configuration using an electrochemical analyzer (Metrohm, μstat-i400). OMIEC thin film coated on a gold electrode served as the working electrode. A platinum wire and an Ag/AgCl were employed as the counter electrode and reference electrode, respectively. EIS measurements were performed at the DC offset potentials of - 0.7 / 0.6V in n/p-type, with a sinusoidal AC amplitude of 10 mV. The frequency range spanned from 10 kHz to 0.1 Hz. The analysis of EIS data was carried out using ZView2 software, the complex impedance data are fitted to Rs(Rp||Q)^[4]^, capacitance was determined using the equation Ceff =[Q Re ^(1-a)^ ] ^1/a^ .

**Grazing Incidence Wide-Angle X-ray Scattering (GIWAXS)**: GIWAXS measurements were carried out using an XEUSS SAXS/WAXS system (Xenocs). Samples were prepared on Au-coated substrates, and the angle of incidence was set to 0.18°. And the solution (P(gTDPPSe): DtFDA: Cell = 20:1:0.1), at a concentration of 10 mg ml^-1^ in chloroform, was spin-coated at a speed of 3000 rmp on the 50 nm Au substrate. The semiconducting layer was then UV cross-linked with a photomask for 6 min under a 280 nm light source and developed it in chloroform for 3 s and blow-drying to complete the patterning. And pure films were prepared by spin coating 10 mg mL^-1^ chloroform solutions at a speed of 3000 rmp on 50 nm Au substrates. Both types of films are about 60-70 nm thick.

**Atomic Force Microscope (AFM):** AFM images are acquired using a Jupiter XR system in tapping mode under ambient conditions. And the solution (P(gTDPPSe): DtFDA: Cell = 20:1:0.1), at a concentration of 10 mg ml^-1^ in chloroform, was spin-coated at a speed of 3000 rmp on the 50 nm Au substrate. The semiconducting layer was then UV cross-linked with a photomask for 6 min under a 280 nm light source and developed it in chloroform for 3 s and blow-drying to complete the patterning. Both types of films were about 60-70 nm thick.

**Electrochemical Quartz Crystal Microbalance (EQCM)**: EQCM measurements were performed using a gold-coated quartz crystal sensor and a quartz crystal microbalance (QCM922A, Princeton Applied Research). The crystal sensor was first tested without loading in both air and electrolyte conditions (0.01 M PBS). Then, the semiconductor solutions (polymer: DtFDA: Cell= 20:1:0.1) (5-10 mg mL^-1^) were spin-coated on the crystal surface at 3000 rpm for 1 min. The crystal sensor with coated film was also tested in both air and electrolyte conditions without biasing. Immediately after the stabilization of the polymer-coated sensor in the electrolyte solution, the system was connected to a PRIMARIUS FS Pro source meter for biasing the film. A three-electrode setup comprised an Ag/AgCl RE, a Pt CE, and the polymer-coated sensor as the WE. For p-type swelling measurements, five cycles of a bias sequence from 0.0 V to +0.8 V and back to 0.0 V were applied to both films. For n-type swelling measurements, the same number of cycles (from 0.0 V to -0.8 V and back to 0.0 V) were applied to the film P(gTDPPSe), with each cycle lasting 100 s. For relative mass change calculation, the Sauerbrey equation was used to convert Δf/_n_ to the change in areal mass (Δm):

$\frac{\Delta m}{A}=-\Delta f_{n}\frac{\rho_{q}V_{q}}{2f_{0}^{2}n}\approx\frac{-\Delta f_{n}}{n}17.9ngcm^{-2}$ (1)

where Δf_n_ is the frequency shift of the nth overtone, A is the sensor active area, *ρ*_q_ is the density of quartz, *V*_q_ is the shear wave velocity in quartz, f_0_ is the fundamental frequency, and n is the overtone number (fixed at 1). Relative mass changes in Figure S13 were calculated as the percentage change in total mass relative to the dry mass:

$Relativemasschange=\frac{m_{\mathrm{total}}-m_{\mathrm{dry}}}{m_{\mathrm{dry}}}\times100\%$ (2)

**vOECT Fabrications:** The vOECTs^[5]^ were fabricated on a Si/300 nm SiO_2_ wafer which was ultrasonic cleaned in isopropyl alcohol and plasma cleaned. First, 3 nm of Cr (rate approximately 0.1-0.3 Å s^−1^) and 150 nm of Au (rate approximately 0.5-1.5 Å s^−1^) were thermally evaporated with a shadow mask as the bottom source electrode. Next, the semiconductor blend chloroform solution (polymer: DtFDA: Cell= 20:1:0.1) was spin-coated on the substrate at 3,000 rpm for 20-30 s. The semiconducting layer was then UV cross-linked with a photomask for 6 min under a 280 nm light source and developed it in chloroform for 3 s and blow-drying to complete the patterning. The top drain electrode (150 nm Au) was then thermally evaporated (rate approximately 0.5-1.5 Å s^−1^) with a shadow mask while maintaining the substrate at a temperature of approximately 5 ºC with a back water-cooling system. The encapsulation solution (DtFDA : Cell= 1:4 ) was spin-coated on the substrate at 5,000 rpm for 20-30 s. And the encapsulation layer was then UV cross-linked with a photomask for 3 min and developed it in chloroform for 3 s and blow-drying. Finally, a droplet (approximately 10 µl, based on the channel area) of 0.01 M PBS was applied on the electrode overlapping area, and an Ag/AgCl electrode was inserted in the droplet acting as the OECT gate electrode. Both types of films were about 60-70 nm thick.

**cOECT Fabrications:** The cOECTs were fabricated on a Si/300 nm SiO_2_ wafer which was ultrasonic cleaned in isopropyl alcohol and plasma cleaned. First, 3 nm of Cr (rate approximately 0.1-0.3 Å s^−1^) and 80 nm of Au (rate approximately 0.5-1.5 Å s^−1^) were thermally evaporated with a shadow mask as the bottom source electrode. Next, a solution of the semiconductor blend in chloroform was spin-coated on the substrate at 3,000 rpm for 20-30 s. The SU8-3002 solution was spin-coated onto the substrate at 2,000 rpm for 60 s. Subsequently, the film was baked at 95 °C for 90 s, exposed using a UV maskless lithography machine (TTT-07-UV Litho-ACA), and post-exposure baked at 95 °C for another 90 s, then the pattern was developed using propylene glycol monomethyl ether acetate (PGMEA). Finally, a droplet (approximately 10 µl, based on the channel area) of 0.01 M PBS was applied on the electrode overlapping area, and an Ag/AgCl electrode was inserted in the droplet acting as the OECT gate electrode. Both types of films were about 60-70 nm thick.

**Inverter Fabrications:** The fabrication process for the OECT-based inverter is similar to that of the vOECTs. First, Au (50 nm) was thermally evaporated onto the glass substrates and patterned with a standard photolithographic/lift-off process. The channel length (L) and width (W) of both the pull-up and pull-down vOECTs are the same. The L of the the pull-up and pull-down vOECTs are 80 µm and 20 µm, respectively. Subsequently, the solution (P(gTDPPSe): DtFDA: Cell = 20:1:0.1), at a concentration of 10 mg ml^-1^ in chloroform, was spin-coated on the substrate. The semiconducting layer was then UV cross-linked with a photomask for 6 min under a 280 nm light source and developed it in chloroform for 3 s and blow-drying to complete the patterning. The top drain electrode (50 nm Au) was then thermally evaporated with a shadow mask while maintaining the substrate at a temperature of approximately 5 ºC with a back water-cooling system. The encapsulation solution (DtFDA : Cell= 1:4) was spin-coated on the substrate at 5,000 rpm for 20-30 s. And the encapsulation layer was then UV cross-linked with a photomask for 3 min and developed it in chloroform for 3 s and blow-drying. Finally, a droplet (approximately 10 µl, based on the channel area) of 0.01 M PBS was applied on the electrode overlapping area, and an Ag/AgCl electrode was inserted in the droplet acting as the inverter gate electrode.

2. Supplementary Figures and Tables

**
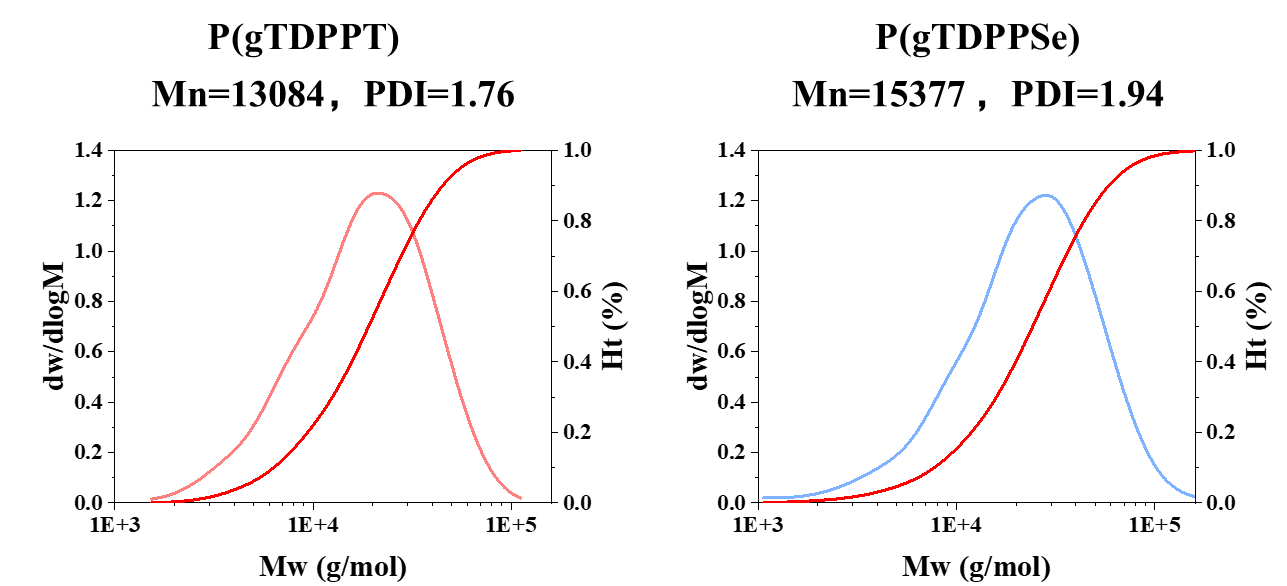
**

**Figure S1**. GPC measurements of P(gTDPPT ) and P(gTDPPSe).


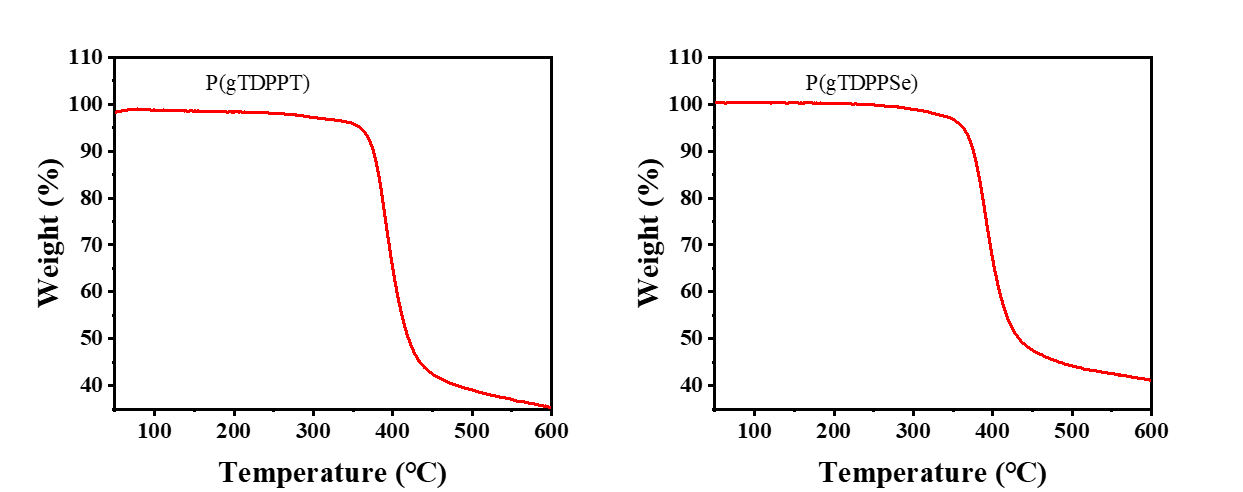


**Figure S2**. TGA measurements of P(gTDPPT) and P(gTDPPSe).


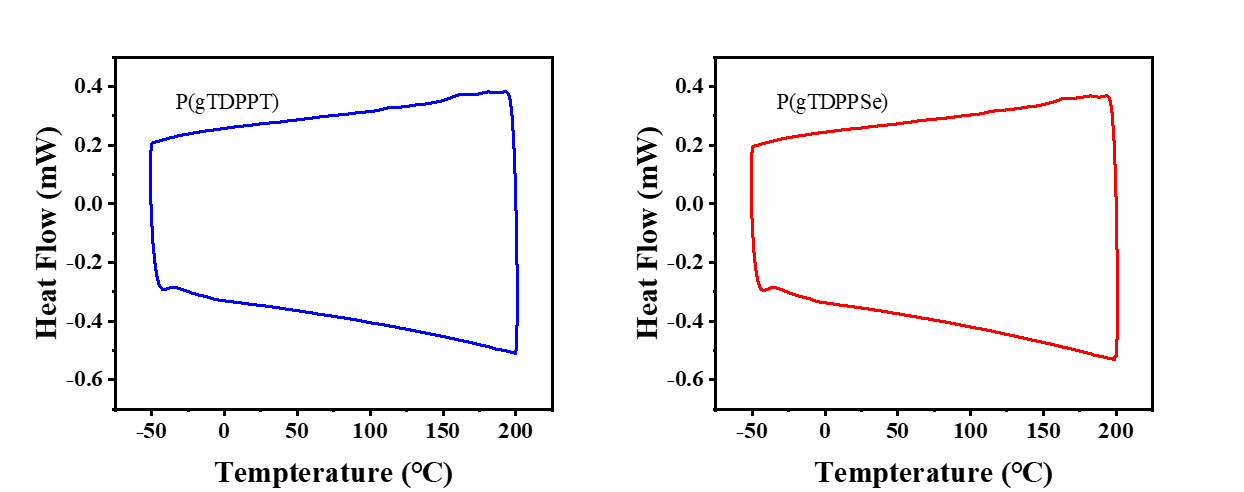


**Figure S3**. DSC measurements of P(gTDPPT) and P(gTDPPSe).


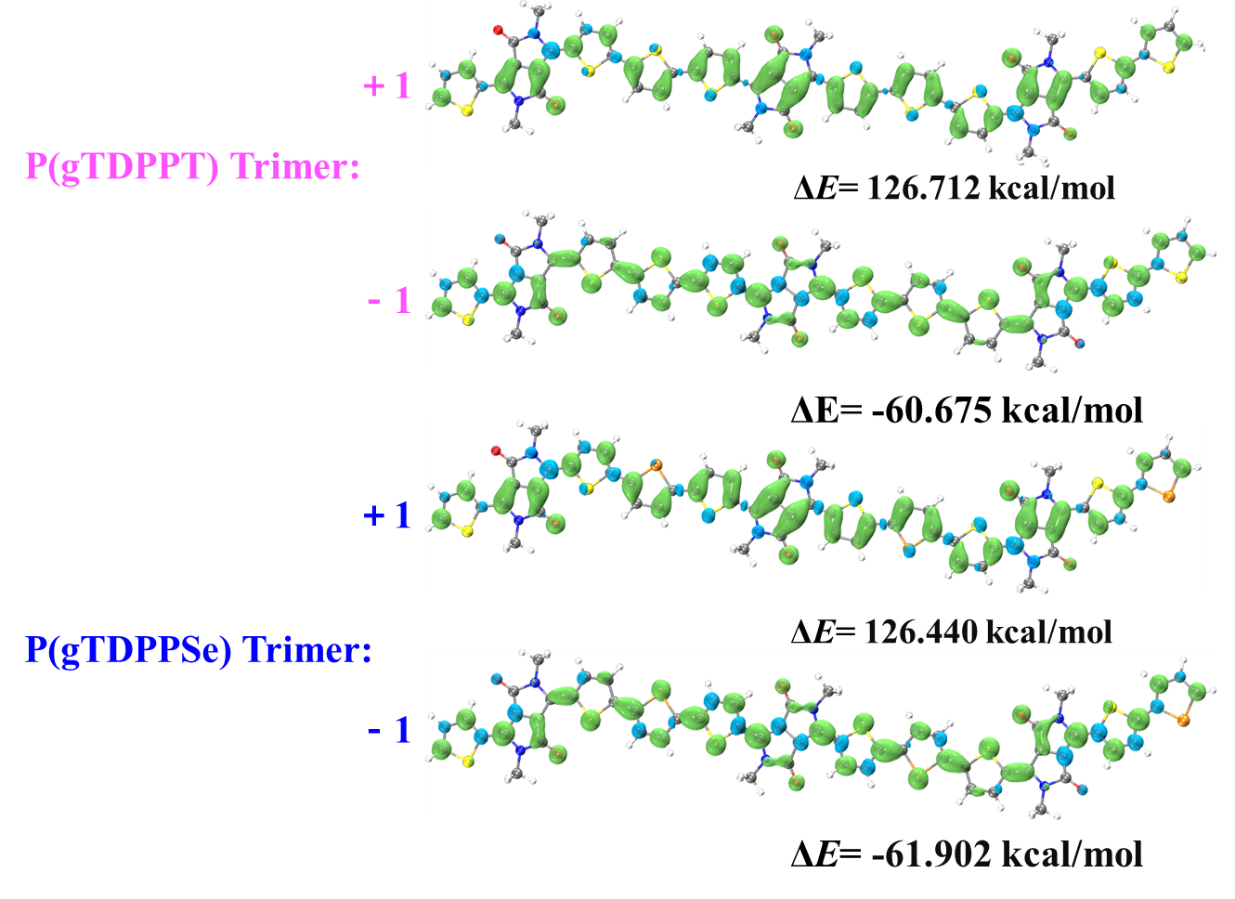


**Figure S4.** Spin density distribution of positively (+) charged and negatively (−) charged polymers of P(gTDPPT) and P(gTDPPSe). ΔE stands for the electronic energy change from the neutral to the charged state.


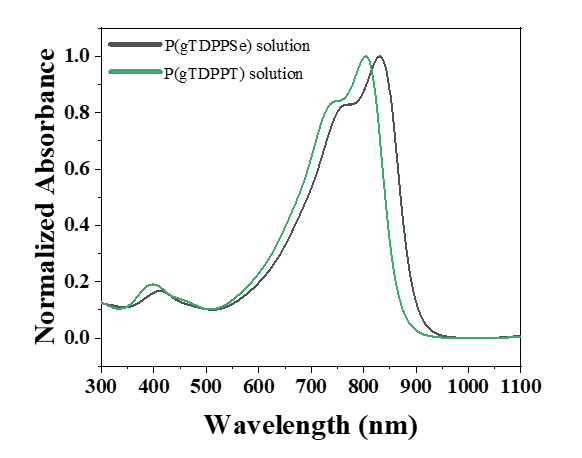


**Figure S5**. Absorption spectra for P(gTDPPT) and P(gTDPPSe) in chloroform solution.


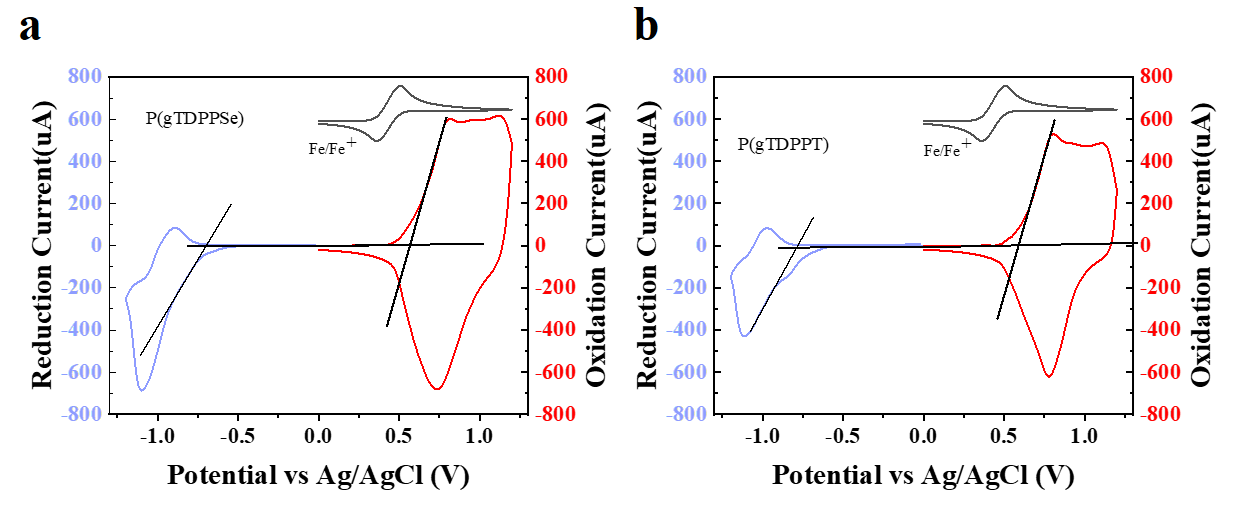


**Figure S6**. CV measurements of P(gTDPPT) and P(gTDPPSe).

**Table S1**. Comparison of the molecular packing parameters of patterned P(gTDPPT) and patterned P(gTDPPSe)

|  | | Lamellar stacking | | | π-π stacking | | | |
| --- | --- | --- | --- | --- | --- | --- | --- | --- |
|  | | q_(100)_  [Å^-1^] | d  [Å] | Lc_(100)_  [Å] | q_(010)_  [Å^-1^] | d  [Å] | Lc_(010)_  [Å] | Packing motif |
| P(gTDPPSe) | In-plane | 0.34 | 18.47 | 29.32 | 1.55 | 4.06 | 4.65 | Face-on |
|  | Out-of-plane |  |  |  | 1.62 | 3.93 | 9.01 |  |
| P(gTDPPT) | In-plane | 0.32 | 19.53 | 27.97 | 1.48 | 4.24 | 3.03 | Face-on |
|  | Out-of-plane |  |  |  | 1.60 | 3.93 | 9.00 |  |


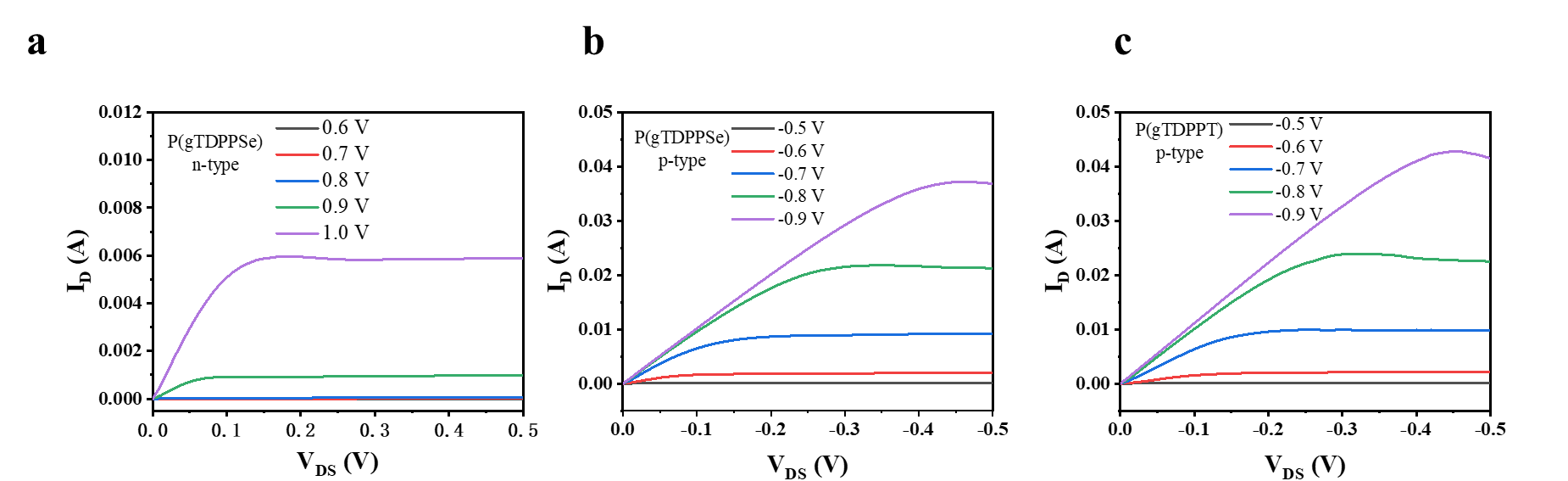


**Figure S7**. Output curves of vOECTs with patterned P(gTDPPSe) and patterned P(gTDPPT): (a) P(gTDPPSe) in n-type, (b) P(gTDPPSe) in p-type and (c) P(gTDPPT) in p-type.


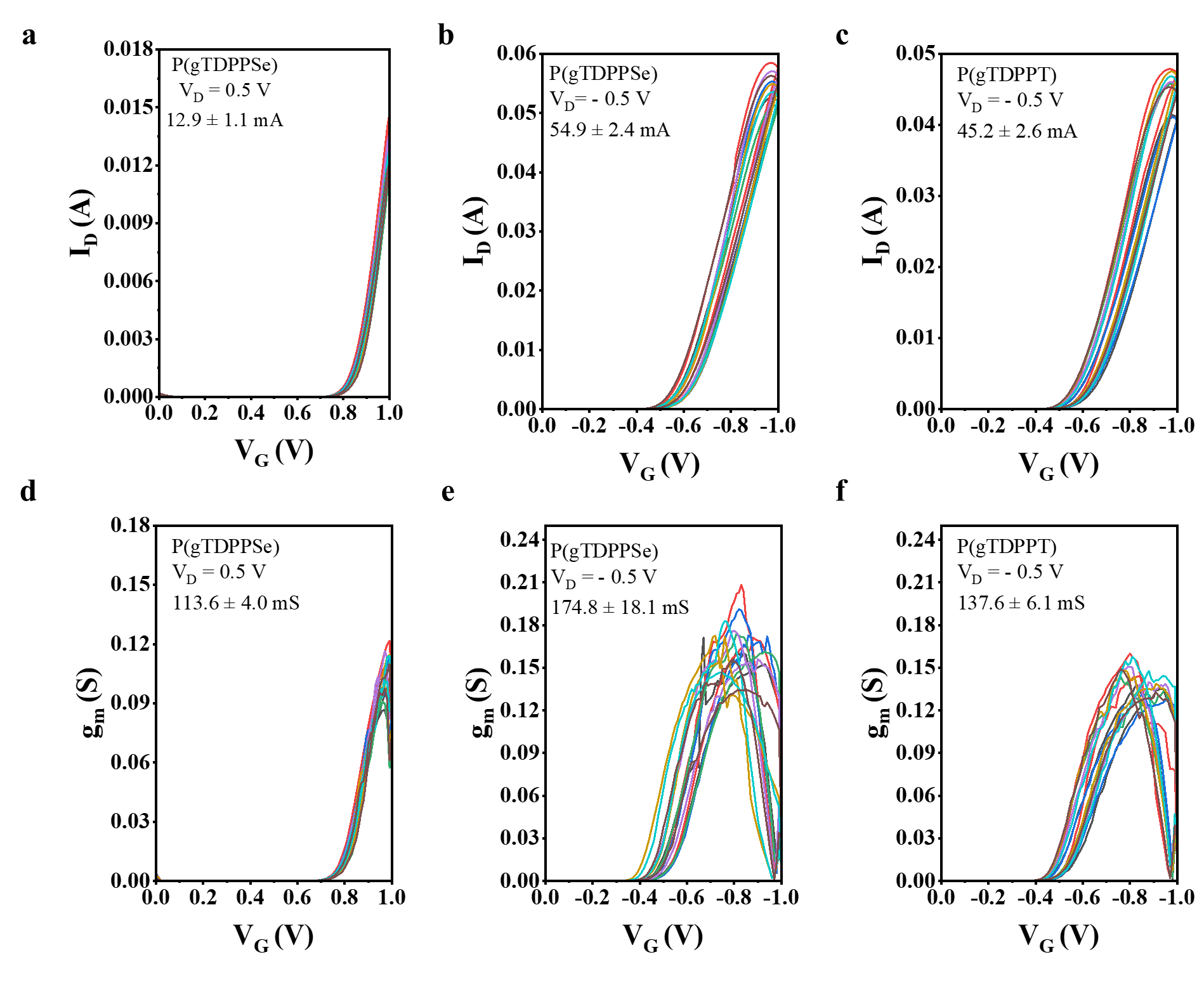


**Figure S8**. Performance of vOECTs. Transfer curves of vOECTs corresponding to eight devices: patterned P(gTDPPSe) in n-type (a, d), patterned P(gTDPPSe) in p-type (b, e), and patterned P(gTDPPT) in p-type (c, f). All vOECTs have the same channel dimensions (W/d = 45 μm / 45 μm).

**
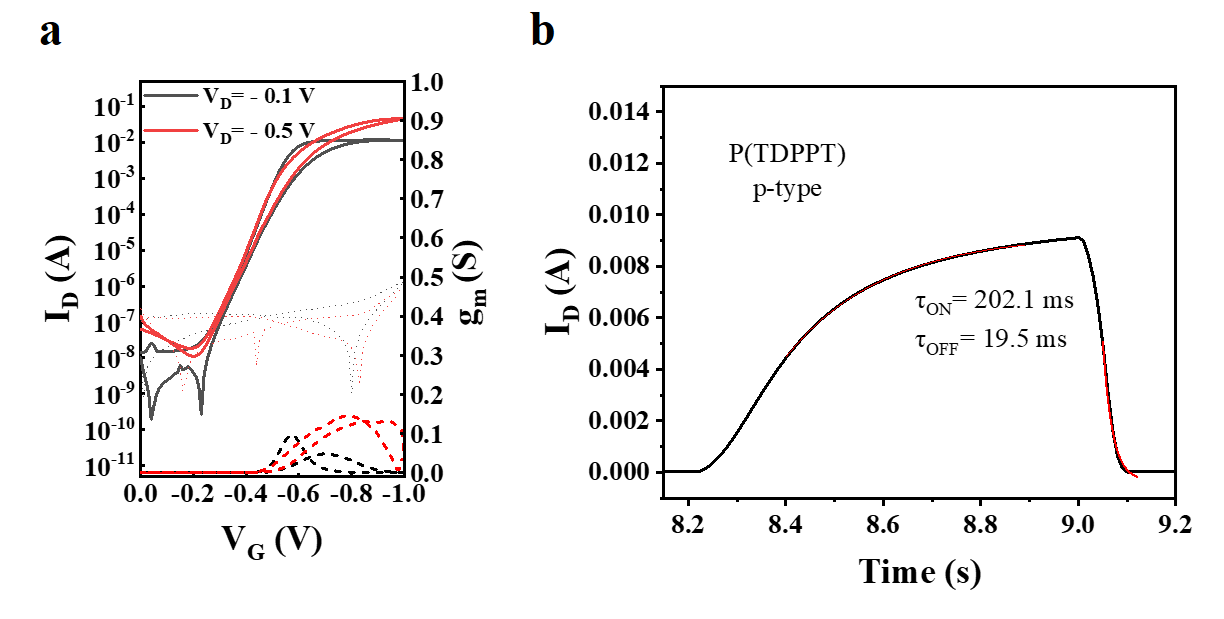
**

**Figure S9.** Output curves of vOECTs with patterned P(gTDPPT) in p-type and switching time for patterned P(gTDPPT) in p-type of *V*_G_ from -0.2 to -1.0 V under constant *V*_D_ of -0.1 V.


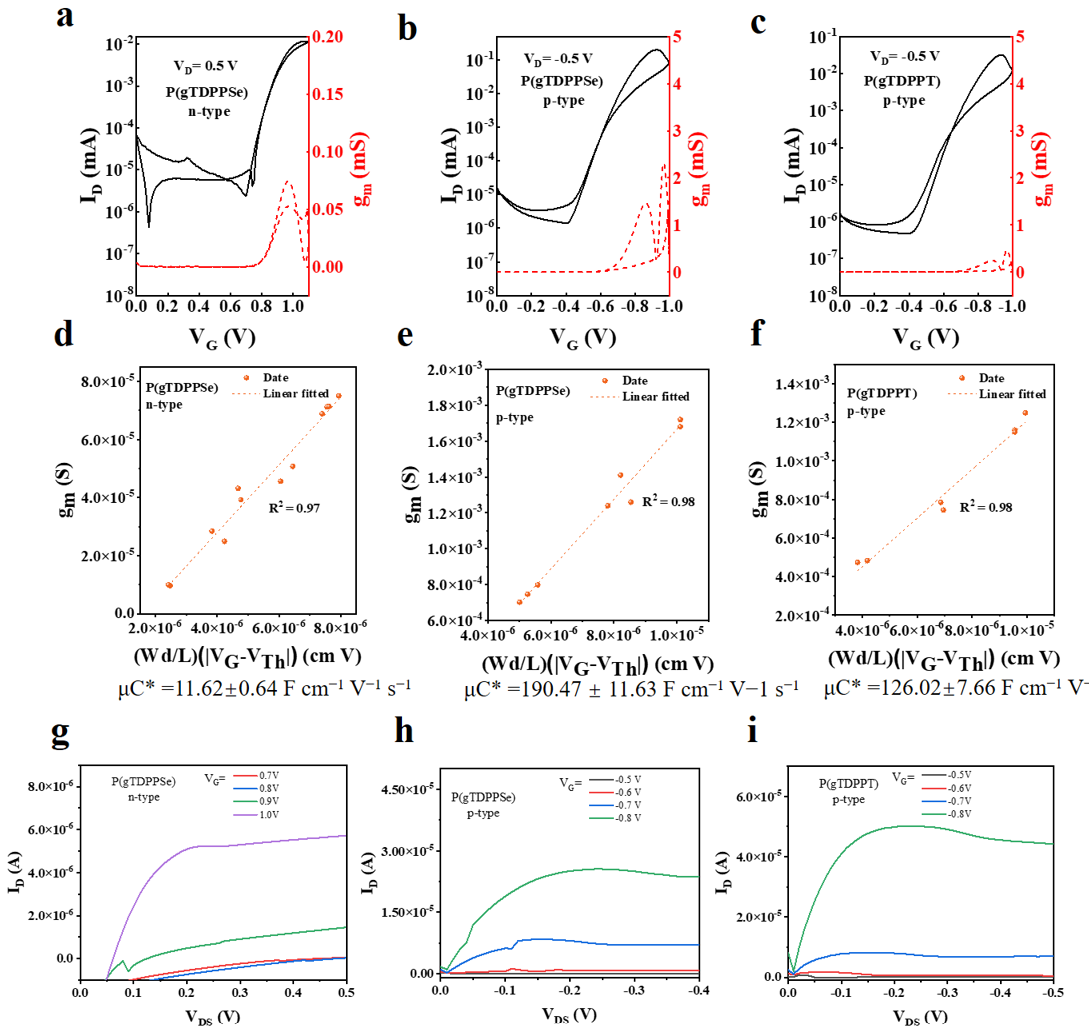


**Figure S10.** Performance of cOECTs. Transfer curves of cOECTs: P(gTDPPSe) in n-type (a), P(gTDPPSe) in p-type (b), and P(gTDPPT) in p-type (c). All cOECTs have the same channel dimensions (W/d = 200 μm / 20 μm). Extraction of μC* from the slope of the transconductance (g_m_) versus (Wd/L) |V_th_ - V_G_| plot for devices with different channel volumes. P(gTDPPSe) in n-type (d), P(gTDPPSe) in p-type (e), and P(gTDPPT) in p-type (f). (g) output curve of P(gTDPPSe) in n-type, (h) output curve of P(gTDPPSe) in p-type, (i) output curve of P(gTDPPT) in p-type.

**Table S2.** Summary of the cOECTs performance parameters of P(gTDPPSe) together with the control polymer P(gTDPPT).

|  | type | d  (nm) | g_m,max_  (mS) | g_m,norm_ ^a)^  (S cm^-1^) | I_ON/OFF_ | *V_th_* ^b)^  (V) | μC* ^c)^  (F V^-1^ cm^-1^ s^-1^) |
| --- | --- | --- | --- | --- | --- | --- | --- |
| P(gTDPPSe) | n | 52 | 0.074 | 1.42 | ~ 10^3^ | 0.80 | 11.62 ± 0.64 |
|  | p | 52 | 1.46 | 28.08 | ~ 10^4^ | -0.68 | 190.47 ± 11.63 |
| P(gTDPPT) | p | 72 | 0.244 | 3.39 | ~ 10^4^ | -0.70 | 126.02 ± 7.66 |

a)Normalized by channel geometry. b) Values obtained by extrapolating the corresponding *I*_D_^1/2^ *V*_G_ plots; c) Extracted from the slope in plots of g_m_ versus (Wd/L) |*V*_th_ -*V*_G_|.

**
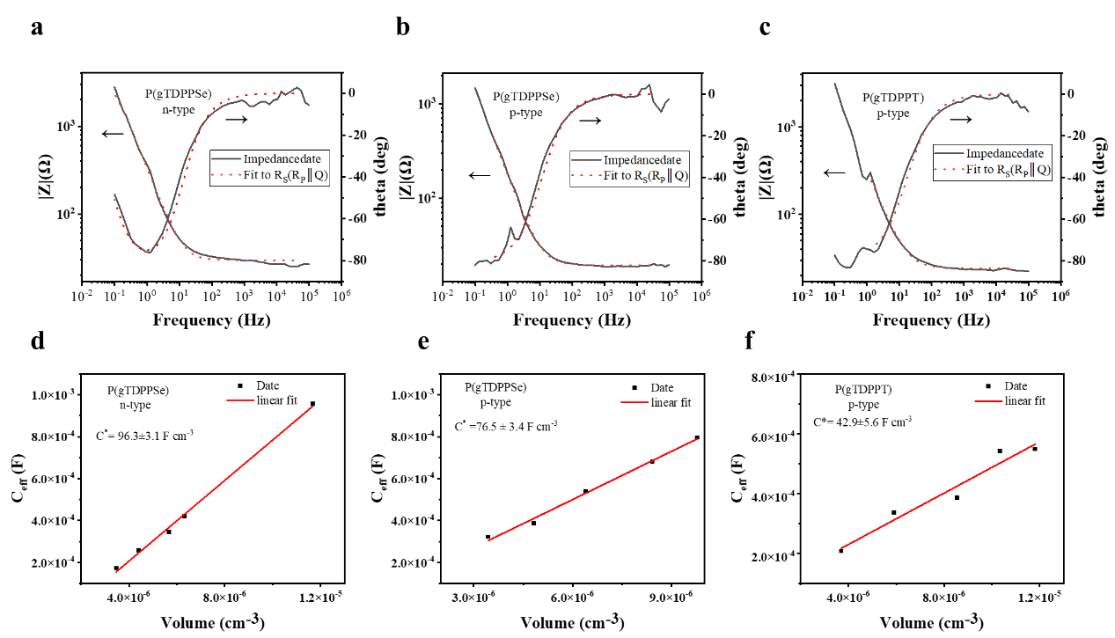
**

**Figure S11**. Electrochemical impedance spectroscopy (EIS) of cross-linked polymer films in 0.01 M PBS: (a) n-type of patterned P(gTDPPSe), (b) p-type of patterned P(gTDPPSe), and (c) p-type of patterned P(gTDPPT). The films were deposited on gold electrodes serving as the working electrode (WE) in 0.01 M PBS. A platinum wire and an Ag/AgCl were employed as the counter electrode and reference electrode, respectively. The complex impedance data are fitted to Rs (Rp||Q) and Rs (Rp1||C) (Rp2||Q). The fitting results are recorded at WE = - 0.7 V in n-type or WE = 0.6 V in p-type. Volume capacitance of a patterned P(gTDPPSe) film in n-type (d), patterned P(gTDPPSe) film in p-type (e) and patterned P(gTDPPT) film in p-type (f) determined from impedance spectroscopy at five different film area.


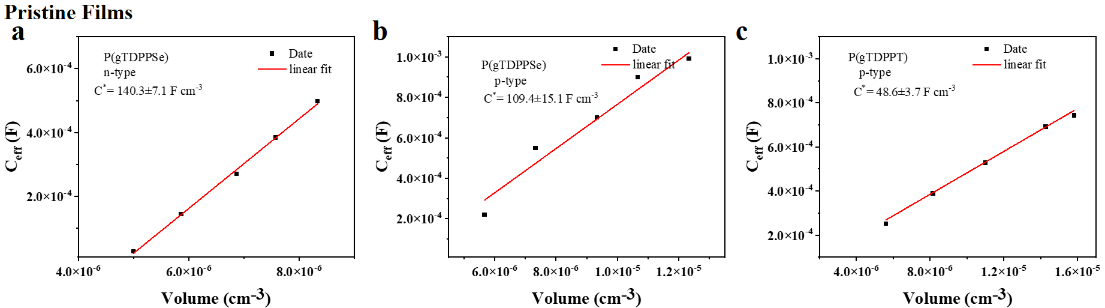


**Figure S12.** Volumetric capacitance of pristine films measured by impedance spectroscopy at five different film areas: (a) n-type P(gTDPPSe), (b) p-type P(gTDPPSe), and (c) p-type P(gTDPPT).


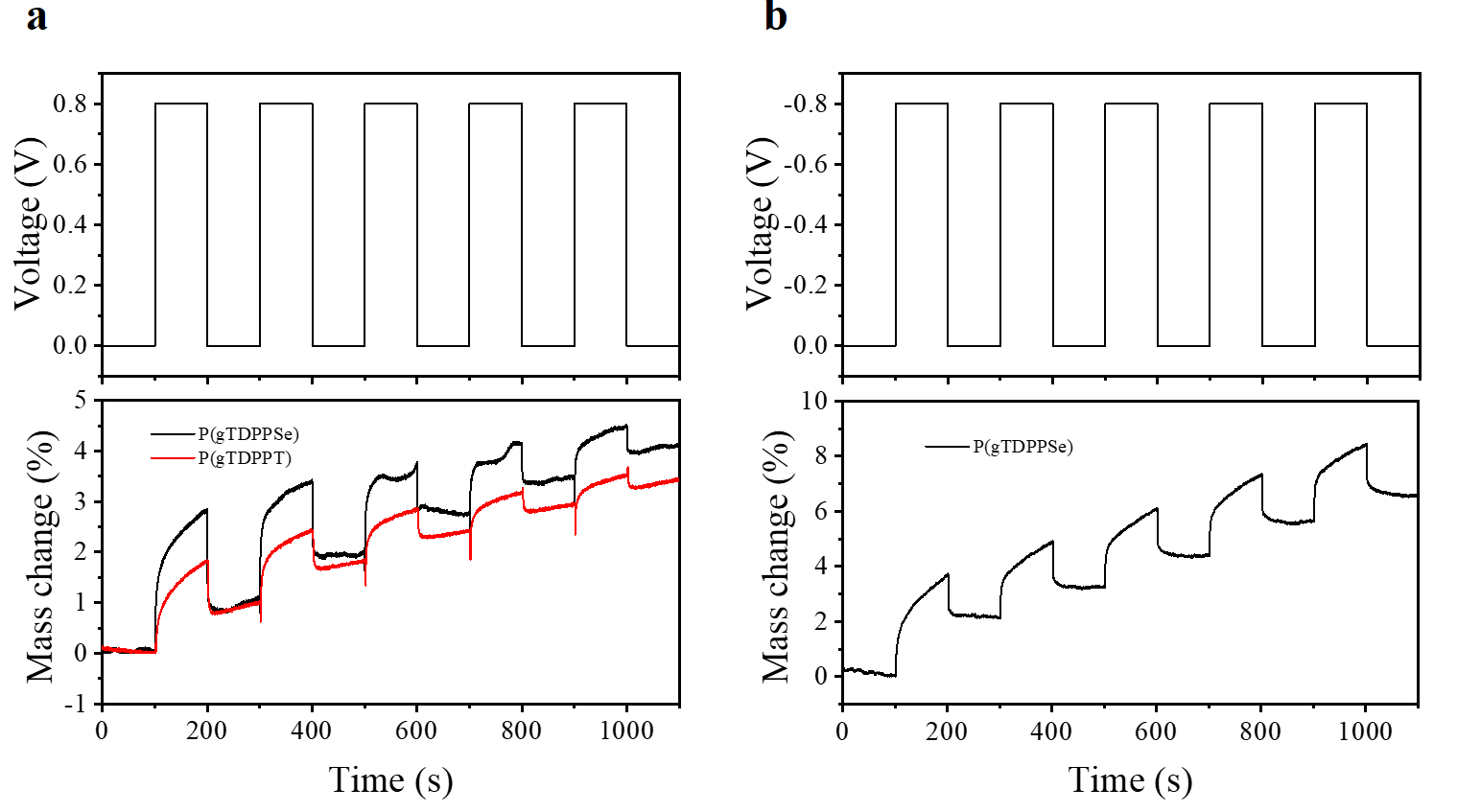


**Figure S13.** a) and b) Relative mass change in response to square-wave potential sweeping, obtained from EQCM


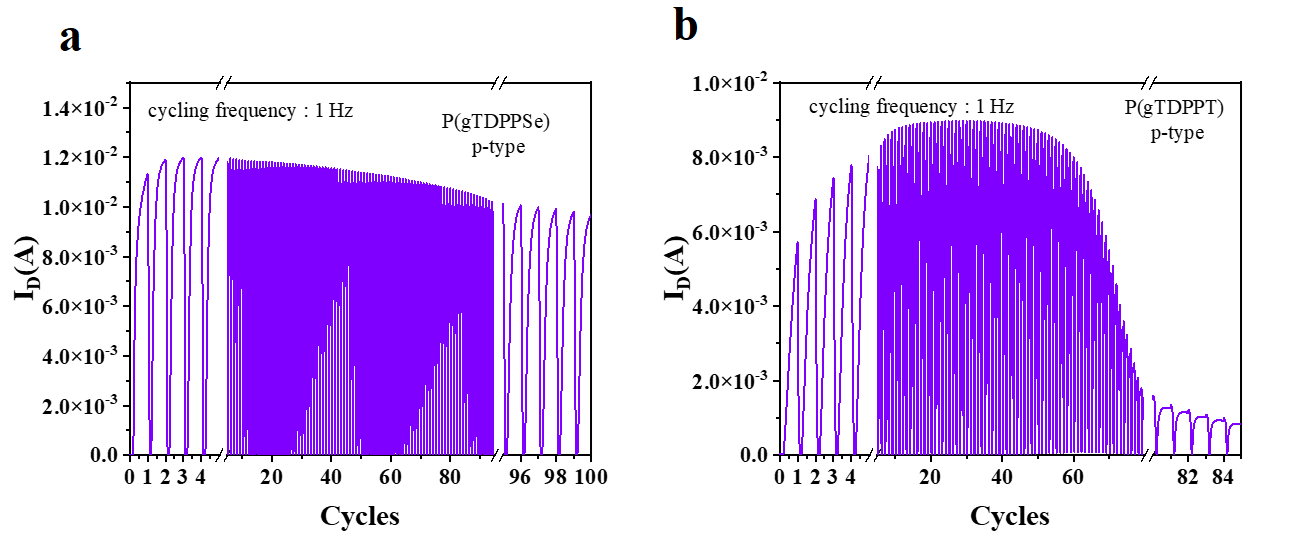


**Figure S14**. Stability for patterned P(gTDPPSe) in p-type (a) and patterned P(gTDPPT) in p-type (b) (W=d=45 μm, L≈65 nm). *V*_G_ from -0.2 to -1.0 V under constant *V*_D_ of -0.1 V.

**
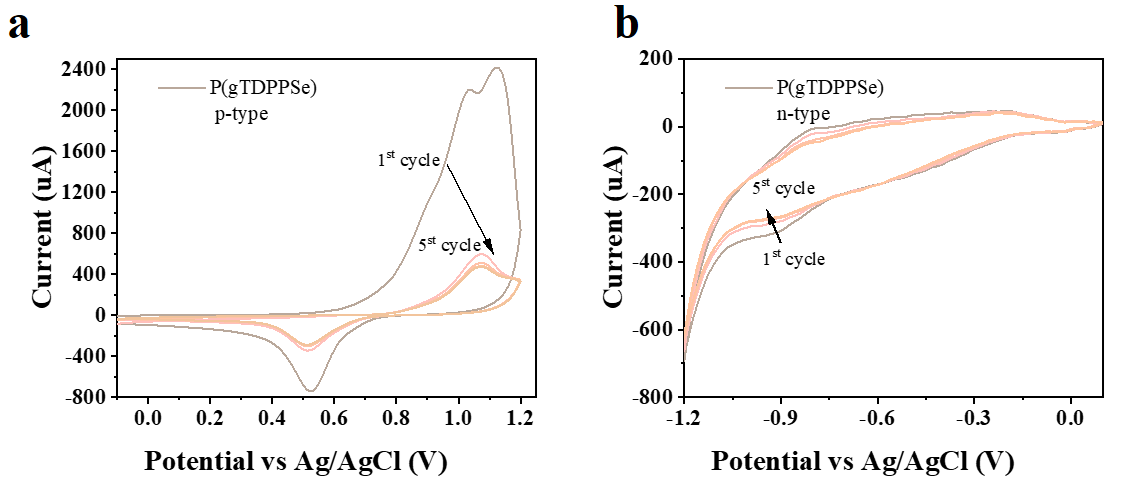
**

**Figure S15.** Five consecutive CV cycles were performed on P(gTDPPSe) films in 0.01 M PBS solution.


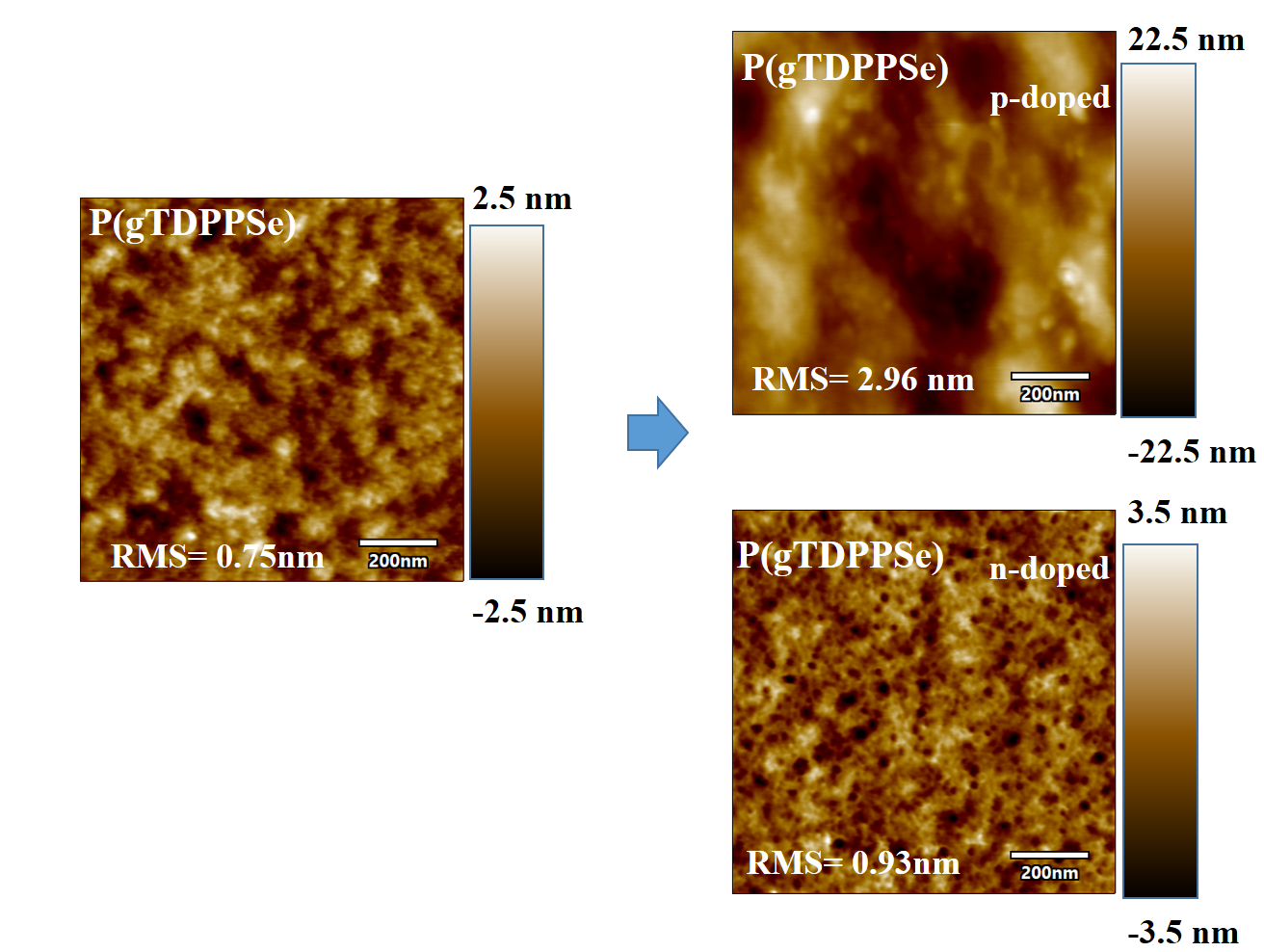


**Figure S16.** Doped patterned P(gTDPPSe) films, with n-type doped 1000 times and p-type doped 100 times in 0.01 M PBS, the doping voltage was consistent with the device operating voltage.

**Table S3**. The summary of inverter performance of ambipolar-based OECTs.

| Polymer | Inverter configuration | *V*_DD_ [V] | Maximum gain | Reference |
| --- | --- | --- | --- | --- |
| **P(gTDPPSe)** | **Ambipolar** | **1.0** | **163** | **This work** |
| p(C4-T2-C0-EG) | Ambipolar | 0.8 | 28 | [6] |
| p(gDPP-V) | Ambipolar | 0.8 | 105 | [7] |
| PrC60MA:p(g2T-TT) = 95:5 (w:w) | Ambipolar | 0.9 | 82 | [8] |
| PBBTL: BBL (3:1) | Ambipolar | 0.6 | 42 | [9] |
| 2DPP-OD-TEG | Ambipolar | 1.4 | 50 | [10] |
| P(gDPP-V-B05) | Ambipolar | 1.4 | 393 | [11] |
| DHF-gTT | Ambipolar | 0.8 | 102 | [12] |
| gIDT-BBT | Ambipolar | 0.9 | 28 | [13] |
| 2gDPP-RD-V | Ambipolar | 0.9 | 135 | [14] |
| P(TII-2FT) | Ambipolar | 0.8 | 809 | [15] |
| P-6O | Ambipolar | 0.9 | 27.4 | [16] |
| PDPP5O-2TzC4 | Ambipolar | 1.0 | 75.4 | [17] |


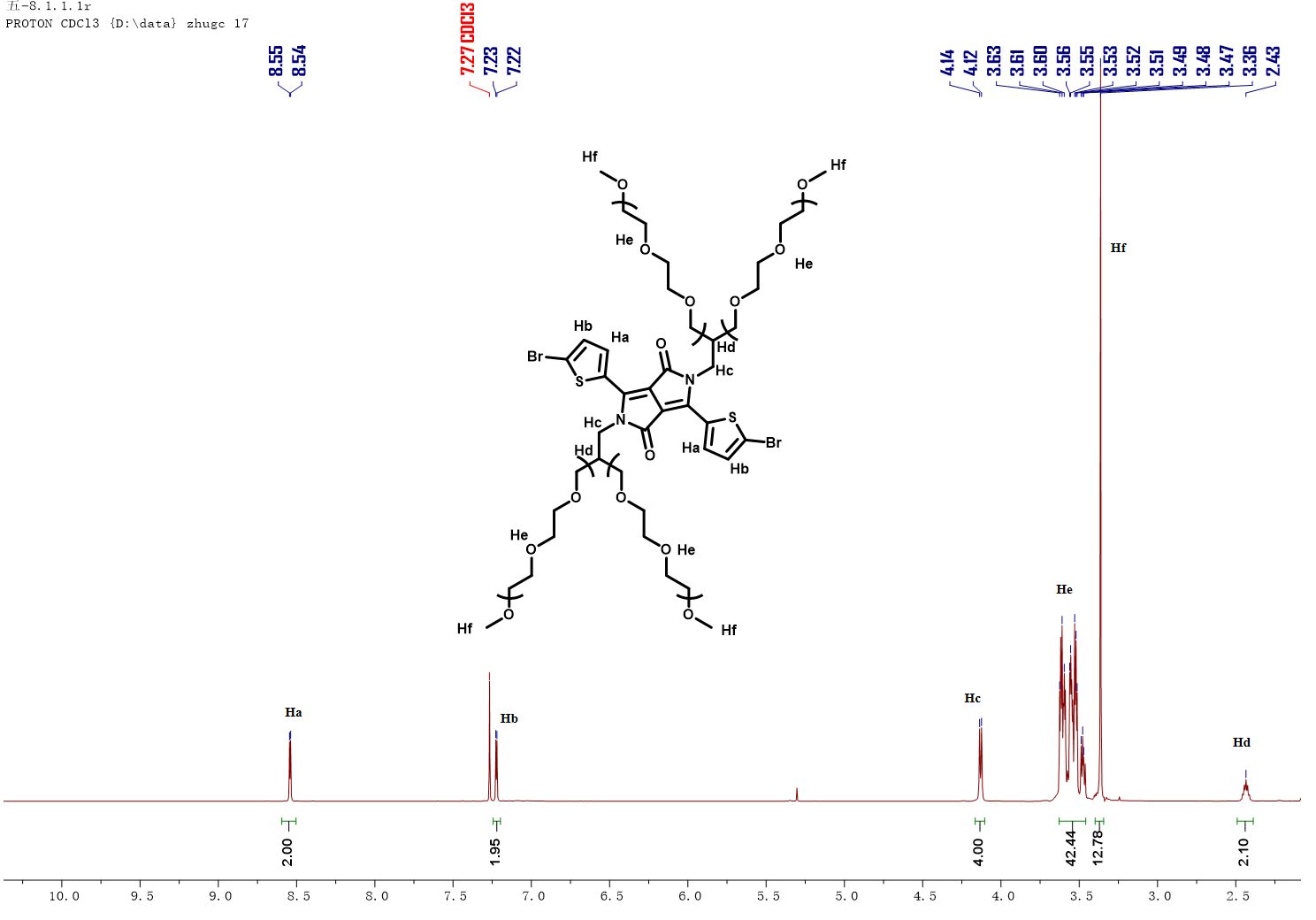


**Figure S17.** The ^1^H NMR spectrum of g-TDPP-Br in CDCl_3_.

**
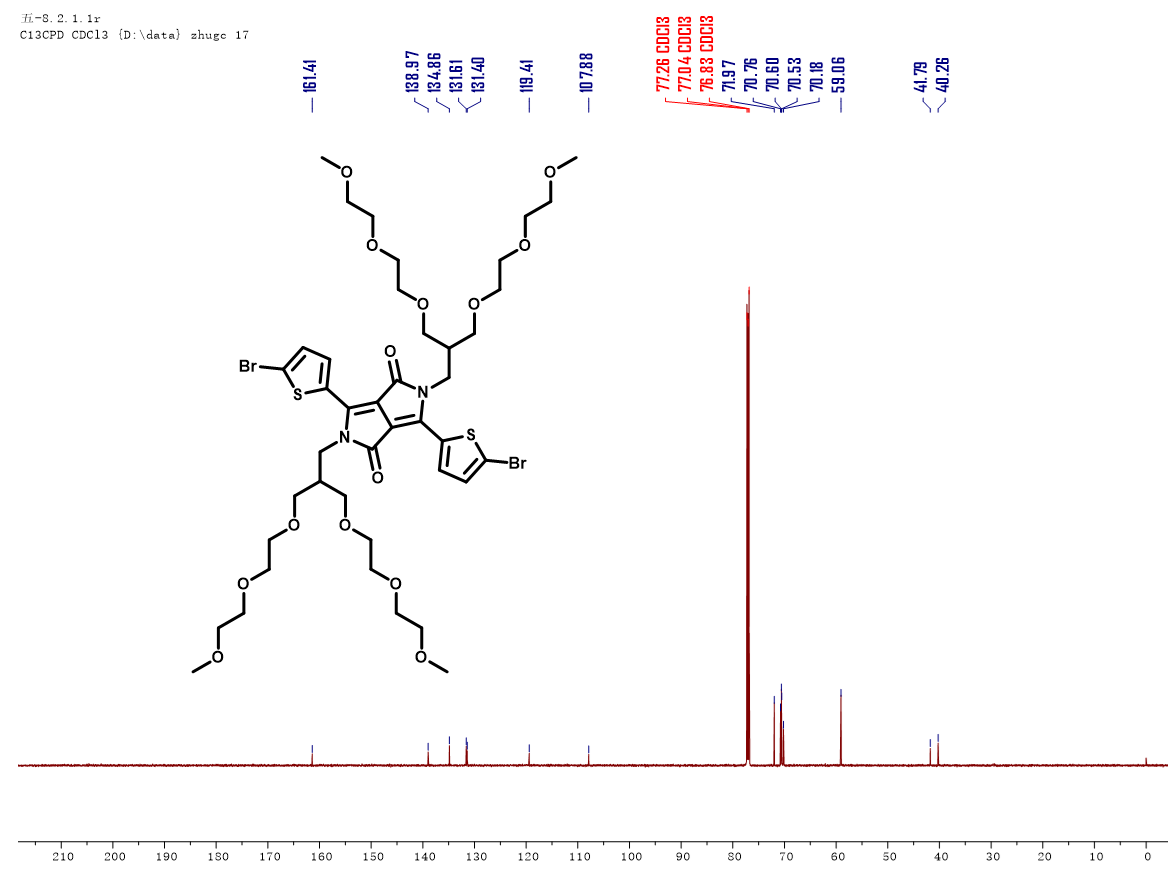
 Figure S18.** The ^13^C NMR spectrum of g-TDPP-Br in CDCl_3_.


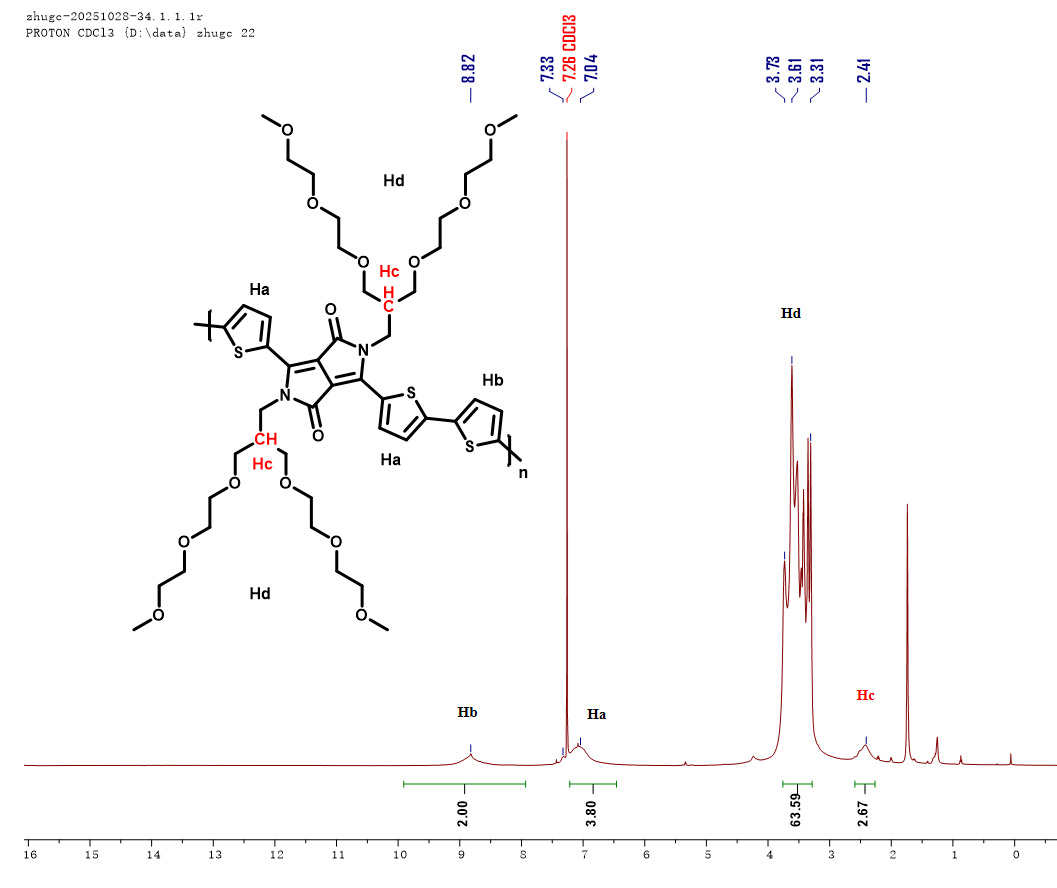


**Figure S19**. The ^1^H NMR spectrum of P(gTDPPT) in CDCl_3_.


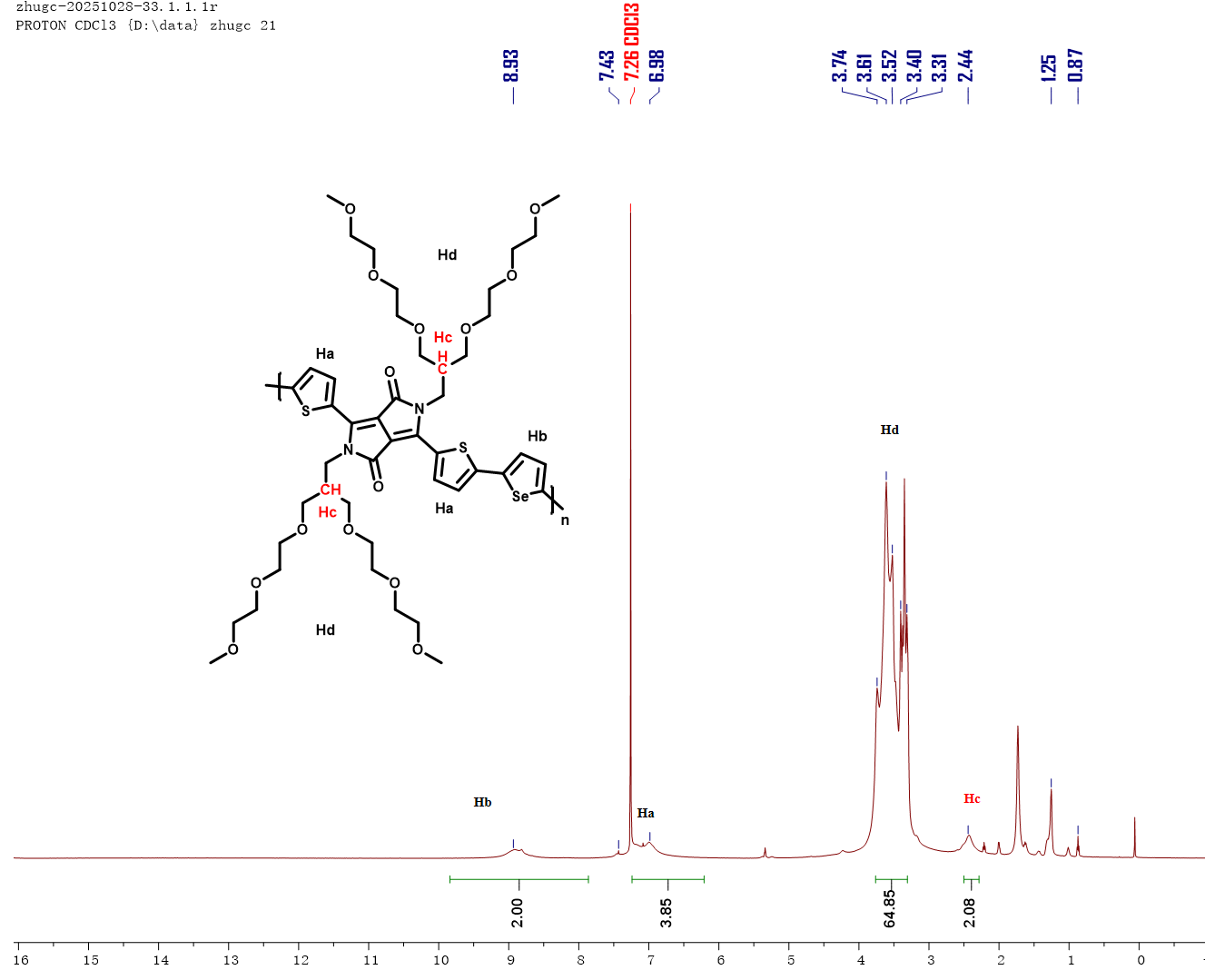


**Figure S20**. The ^1^H NMR spectrum of P(gTDPPSe) in CDCl_3_.

3. Supplementary References.

[1] X. Chen, Z. Zhang, Z. Ding, J. Liu, L. Wang, "Diketopyrrolopyrrole-based Conjugated Polymers Bearing Branched Oligo(Ethylene Glycol) Side Chains for Photovoltaic Devices*" Angewandte Chemie International Edition* **55**, (2016): 10376-10380.

[2] K. Feng, W. Shan, J. Wang, J.-W. Lee, W. Yang, W. Wu, Y. Wang, B. J. Kim, X. Guo, H. Guo, "Cyano-Functionalized n-Type Polymer with High Electron Mobility for High-Performance Organic Electrochemical Transistors*" Advanced Materials* **34**, (2022): 2201340.

[3] M. Moser, A. Savva, K. Thorley, B. D. Paulsen, T. C. Hidalgo, D. Ohayon, H. Chen, A. Giovannitti, A. Marks, N. Gasparini, A. Wadsworth, J. Rivnay, S. Inal, I. McCulloch, "Polaron Delocalization in Donor–Acceptor Polymers and its Impact on Organic Electrochemical Transistor Performance*" Angewandte Chemie International Edition* **60**, (2021): 7777-7785.

[4] H. Sun, M. Vagin, S. Wang, X. Crispin, R. Forchheimer, M. Berggren, S. Fabiano, "Complementary Logic Circuits Based on High-Performance n-Type Organic Electrochemical Transistors*" Advanced Materials* **30**, (2018): 1704916.

[5] W. Huang, J. Chen, Y. Yao, D. Zheng, X. Ji, L.-W. Feng, D. Moore, N. R. Glavin, M. Xie, Y. Chen, R. M. Pankow, A. Surendran, Z. Wang, Y. Xia, L. Bai, J. Rivnay, J. Ping, X. Guo, Y. Cheng, T. J. Marks, A. Facchetti, "Vertical organic electrochemical transistors for complementary circuits*" Nature* **613**, (2023): 496-502.

[6] R. B. Rashid, X. Ji, J. Rivnay, "Organic electrochemical transistors in bioelectronic circuits*" Biosensors and Bioelectronics* **190**, (2021): 113461.

[7] S. Cong, J. Chen, M. Xie, Z. Deng, C. Chen, R. Liu, J. Duan, X. Zhu, Z. Li, Y. Cheng, W. Huang, I. McCulloch, W. Yue, "Single ambipolar OECT–based inverter with volatility and nonvolatility on demand*" Science Advances* **10**: eadq9405.

[8] E. Stein, O. Nahor, M. Stolov, V. Freger, I. M. Petruta, I. McCulloch, G. L. Frey, "Ambipolar blend-based organic electrochemical transistors and inverters*" Nature Communications* **13**, (2022): 5548.

[9] X. Wu, T. L. D. Tam, S. Chen, T. Salim, X. Zhao, Z. Zhou, M. Lin, J. Xu, Y.-L. Loo, W. L. Leong, "All-Polymer Bulk-Heterojunction Organic Electrochemical Transistors with Balanced Ionic and Electronic Transport*" Advanced Materials* **34**, (2022): 2206118.

[10] J. J. Samuel, A. Garudapalli, A. A. Mohapatra, C. Gangadharappa, S. Patil, N. P. B. Aetukuri, "Single-Component CMOS-Like Logic using Diketopyrrolopyrrole-Based Ambipolar Organic Electrochemical Transistors*" Advanced Functional Materials* **31**, (2021): 2102903.

[11] Y. Wang, J. Tan, H. Hou, H. Maricherla, M. K. Ravva, X. Zhu, R. Liu, J. He, Y. Lin, I. McCulloch, Z. Li, W. Yue, "High Performing Ambipolar Organic Electrochemical Transistors and Solid-State Inverters Enabled by Hydrophilic/Hydrophobic Side Chains Integration*" Advanced Materials* , (2025), DOI: 10.1002/adma.202515186.

[12] G. Qi, M. Wang, S. Wang, S. Zhang, X. Teng, H. Bai, B. Wang, C. Zhao, W. Su, Q. Fan, W. Ma, "High-Performance, Single-Component Ambipolar Organic Electrochemical Transistors with Balanced n/p-Type Properties for Inverter and Biosensor Applications*" Advanced Functional Materials* **35**, (2025): 2413112.

[13] Y. Sun, Y. Lan, M. Li, W. Feng, M. Xie, Y. Lai, W. Li, Y. Cheng, J. Chen, W. Huang, L.-W. Feng, J. Ding, "Indacenodithiophene-based single-component ambipolar polymer for high-performance vertical organicelectrochemical transistors and inverters*" Aggregate* **5**, (2024): e577.

[14] L. Lan, Y. Wang, X. Zhu, I. McCulloch, W. Yue, "Ultrathin-Film Small Molecule Mixed Conductors Exhibiting Ion-Tunable Ambipolarity for High-Performance Organic Electrochemical Transistors and Multivalued Logic Inverters*" Advanced Materials* **37**, (2025): 2501041.

[15] G.-Y. Ge, J. Xu, X. Wang, W. Sun, M. Yang, Z. Mei, X.-Y. Deng, P. Li, X. Pan, J.-T. Li, X.-Q. Wang, Z. Zhang, S. Lv, X. Dai, T. Lei, "On-site biosignal amplification using a single high-spin conjugated polymer*" Nature Communications* **16**, (2025): 396.

[16] T. Pan, X. Jiang, E. R. W. van Doremaele, J. Li, T. P. A. van der Pol, C. Yan, G. Ye, J. Liu, W. Hong, R. C. Chiechi, Y. v. de Burgt, Y. Zhang, "Over 60 h of Stable Water-Operation for N-Type Organic Electrochemical Transistors with Fast Response and Ambipolarity*" Advanced Science* **11**, (2024): 2400872.

[17] J. Li, Z. Li, J. Huang, J. Su, P. Xu, S. Wang, P. Zhang, Y. Tian, T. Pan, J. Liu, J. Li, G. Ye, R. C. Chiechi, Y. Zhang, W. Hong, "High-Performance Ambipolar Organic Electrochemical Transistors Based on Diketopyrrolopyrrole-Dialkoxybithiazole Conjugated Polymers for Single-component Inverters*" Advanced Science* (2026), DOI: 10.1002/advs.202520003.
